# Differences in the intrinsic chondrogenic potential of equine umbilical cord matrix and cord blood mesenchymal stromal/stem cells for cartilage regeneration

**DOI:** 10.1038/s41598-018-28164-9

**Published:** 2018-09-14

**Authors:** Rodolphe Rakic, Bastien Bourdon, Magali Demoor, Stéphane Maddens, Nathalie Saulnier, Philippe Galéra

**Affiliations:** 1 Normandie Univ, Unicaen, Biotargen, Caen, 14000 Caen France; 2 Vetbiobank, 69280 Marcy l’Etoile, France

**Keywords:** Mesenchymal stem cells, Cartilage

## Abstract

Umbilical cord blood mesenchymal stromal/stem cells (UCB-MSCs) and umbilical cord matrix MSCs (UCM-MSCs) have chondrogenic potential and are alternative sources to standard surgically derived bone marrow or adipose tissue collection for cartilage engineering. However, the majority of comparative studies explore neonatal MSCs potential only on ISCT benchmark assays accounting for some bias in the reproducibility between *in vitro* and in clinical studies. Therefore, we characterized equine UCB-MSCs and UCM-MSCs and investigated with particular attention their chondrogenesis potential in 3D culture with BMP-2 + TGF-ß1 in normoxia or hypoxia. We carried out an exhaustive characterization of the extracellular matrix generated by both these two types of MSCs after the induction of chondrogenesis through evaluation of hyaline cartilage, hypertrophic and osteogenic markers (mRNA, protein and histology levels). Some differences in hypoxia sensitivity and chondrogenesis were observed. UCB-MSCs differentiated into chondrocytes express an abundant, dense and a hyaline-like cartilage matrix. By contrast, despite their expression of cartilage markers, UCM-MSCs failed to express a relevant cartilage matrix after chondrogenic induction. Both MSCs types also displayed intrinsic differences at their undifferentiated basal status, UCB-MSCs expressing higher levels of chondrogenic markers whereas UCM-MSCs synthesizing higher amounts of osteogenic markers. Our results suggest that UCB-MSCs should be preferred for *ex-vivo* horse cartilage engineering. How those results should be translated to *in vivo* direct cartilage regeneration remains to be determined through dedicated study.

## Introduction

Mesenchymal stromal/stem cells (MSCs) are an attractive source of stem cells for regenerative medicine, due to their plasticity, trophic factors secretion and immunomodulatory properties^1^. Considerable effort and financial resources have been invested in research into MSCs and their clinical use both in human and veterinary medicine. Bone marrow MSCs (BM-MSCs) and adipose-derived MSCs (AD-MSCs) are the most frequently investigated sources of MSCs. However, the use of adult MSCs is limited by the need for invasive procedures to collect the cells, together with their lower proliferation potential, intrinsic and extrinsic factors sensitivity, and the impact of aging on their biological properties^2–4^. To circumvent these limitations, new sources have been explored to isolate MSCs. In particular, neonatal tissues-derived MSCs harbor several advantages over adult MSCs: the procurement procedure is painless and noninvasive, and these cells have a higher proliferation capacity than adult MSCs^5^. Moreover, neonatal MSCs seem to express pluripotent markers and do not display the same effects of aging observed in cells from adult donors^6^. The absence of ethical constraints and the considerable potential of neonatal MSCs for expansion have thus raised new hopes for the development of stem cell therapies, with the possibility of industrialization in good manufacturing practices (GMP) conditions^7^.

Various neonatal tissues have been described to isolate MSCs in horse, including umbilical cord blood (UCB-MSCs)^8–11^, umbilical cord matrix (UCM-MSCs), commonly referred as “Wharton’s jelly”^12^, amniotic tissue^13^, or placenta^14^. Such cells meet the standards recommended by the International Society of Cellular Therapy (ISCT) for MSCs^15^, including plastic adherence, multilineage differentiation potential, and a particular immunophenotype. Nonetheless, these standards have been established following a scientific consensus based on results mainly obtained from human BM-MSCs, and do not predict the clinical potency of the cells^16^.

This is particularly true when considering the use of MSCs to develop a tissue-engineered cartilage substitute. It is widely accepted that neonatal MSCs are able to differentiate toward the chondrogenic lineage *in vitro*, but the choice of the most suitable MSCs source for cartilage tissue-engineered remains difficult. In the equine model, different groups reported the production of a cartilage neosubstitute using bone marrow-derived MSCs cultured in 3 dimensional (3D) models^17–19^. Nevertheless, only few studies explored the chondrogenic differentiation ability of neonatal equine MSCs. More informations were obtained from researches performed on human neonatal MSCs. While the chondrogenic ability of UCB-MSCs has been largely reported^20,21^, less consistent informations were available on the chondrogenic differentiation of UCM-MSCs, with divergent results^22–26^. This demonstrates the need to develop further experimental assays that may help to define the better source for cartilage tissue engineering both in human and horse models.

Chondrogenesis is a multiparametric differentiation process dependent on the cellular microenvironment^27^. The commitment of cells to this lineage requires particular conditions, and development of the chondrogenic lineage is closely related to hypertrophic chondrocyte maturation and osteogenesis, as during endochondral ossification in embryonic development^28^. The successful differentiation of MSCs into chrondrocytes is dependent on 1- a three-dimensional (3D) network^29^, 2- a particular cocktail of chondrogenic growth factors^30^, 3- an hypoxic environment^31^. A subtle regulation of all these parameters is required to produce a specific hyaline cartilage extracellular matrix (ECM).

We have previously shown that human UCB-MSCs cultured in a three-dimensional (3D) sponge of type I/III collagen with transforming growth factor 1 (TGF-ß1) and bone morphogenetic protein 2 (BMP-2) can generate a hyaline-like extracellular matrix of potential value for tissue engineering^32^. To gather further information about the potential of neonatal MSCs for cartilage tissue generation, we carry on new investigations with equine UCM- and UCB-MSCs and compare their ability to generate a hyaline like cartilage. Following a systematic characterization of the cells according to the ISCT recommendations, UCM- and UCB-MSCs were cultured under a standardized chondrogenic differentiation process, including a 3D cell culture model and BMP-2/ TGF-ß1 (BT) stimulation for 28 days. The influence of hypoxic condition was also evaluated for the first time on equine neonatal MSCs lineage commitment. Our data showed significant differences in the capacity of UCB- and UCM-MSCs to differentiate toward the chondrogenic lineage. Furthermore, our results suggest that, unlike BM-MSCs, low oxygen tension do not improve the chondrogenic differentiation of neonatal MSCs. These data could help to design strategies for improving cartilage tissue engineering.

## Results

### UCM- and UCB-MSCs characterization

**Figure 1:**
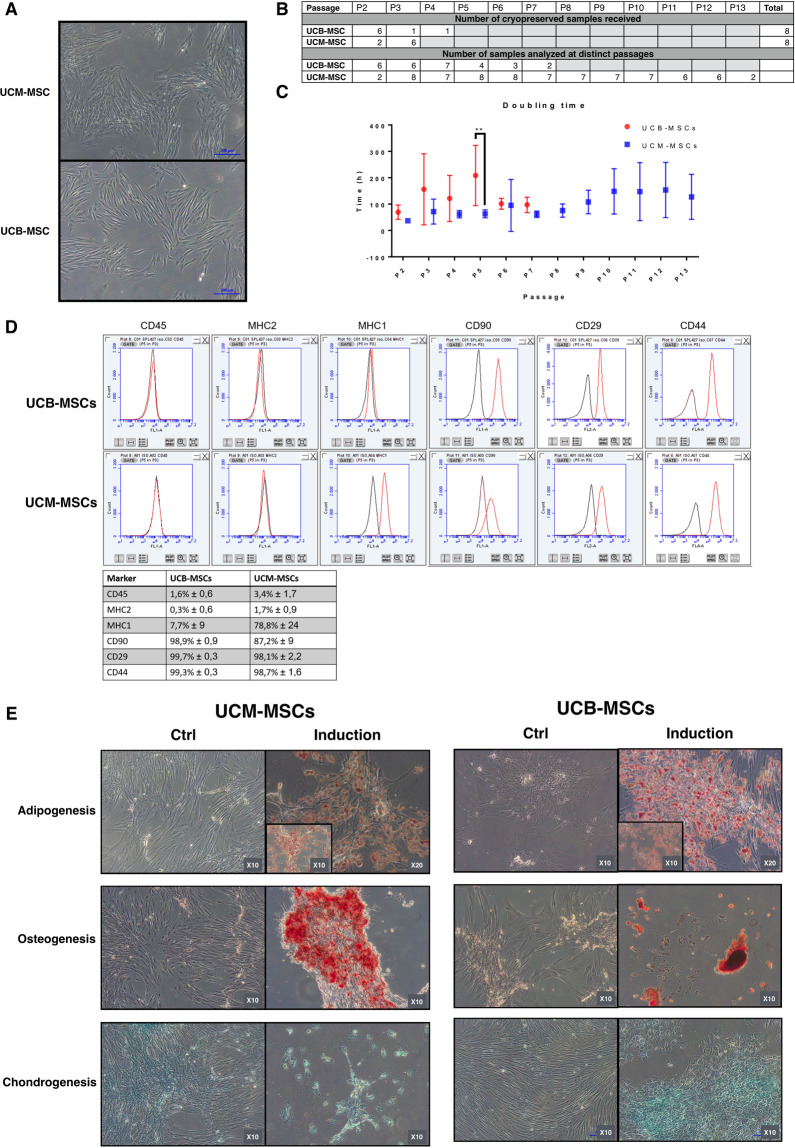
UCM-MSCs have a higher proliferation rate than UCB-MSCs. (**A**) Morphological aspect of UCM-MSCs and UCB-MSCs during monolayer cell culture at P3 observed by phase-contrast microscopy (magnification × 10). (**B**) Samples characteristics. This table recapitulates the number of independent cryopreserved UCM- and UCB-MSCs vials received for analysis, and the number of samples evaluated for each passage. (**C**) Proliferation characteristics of UCM- and UCB-MSCs upon successive passages. This graph represents the doubling time (h) of UCB-MSCs and UCM-MSCs cultured up to P13. Samples with doubling time above 400 h were systematically excluded from the representation, these samples being considered as non-proliferating. A statistical analysis was realized to compare the proliferation potential of UCM- and UCB-MSCs using multiple comparison two-way ANOVA test (**p < 0.01) when the number of samples was appropriated (from P2 to P7). (**D**) Flow cytometry graph analysis of representative samples of UCM-MSCs and UCBM-MSCs. Recapitulative table of mean values and standard deviation is presented for CD-45, MHC-1, MHC-2, CD-90, CD-29 and CD-44. (**E**) UCB-MSCs and UCM-MSCs multilineage évaluation. UCM-MSCs and UCB-MSCs were induced in adipogenic, osteogenic and chondrogenic lineage by using specific induction media. ECM mineralization was demonstrated by alizarin red S staining. Lipid droplets in the cytoplasm are highlighted by oil red O staining and sulfated matrix observed during chondrogenesis was evidenced by alcian blue staining. A representative example from seven samples for UCM-MSCs and five samples for UCB-MSCs are shown (magnification: ×10 and ×20).

UCM- and UCB-MSCs display a fibroblastic-like appearance, hallmark of MSCs populations (Fig. 1A). Growth rates of both MSCs populations were investigated over 15 consecutive passages *in vitro*, when feasible. Our results showed that UCM-MSCs have a higher proliferation capacity than UCB-MSCs (Fig. 1B,C). Only two samples of UCB-MSCs expanded during at least seven passages. Immunophenotyping of both UCM- and UCB-MSCs showed a similar profile for the following markers: CD90+/CD44+/CD29+/MHC2−/CD45− (Fig. 1D). The multipotency of UCM- and UCB-MSCs was assessed by inducing differentiation into adipocytes, osteoblasts and chondrocytes in monolayer cultures in specific differentiation media (Fig. 1E). Both UCM- and UCB-MSCs displayed commitment into the adipogenic lineage, as assessed by the presence of a large number of lipid droplets stained with O red oil. Osteogenic differentiation was evaluated by staining the calcium phosphate deposits with Alizarin red S. Intense labeling was observed in UCM-MSCs stimulated with the osteogenic medium, while results were more heterogeneous with UCB-MSCs, as several cell lines detach from their support during the induction, impeding a valuable interpretation of the results. Chondrogenic differentiation was assessed by Alcian blue staining after 21 days of differentiation. UCB-MSCs express sulfated ECM colored by Acian blue. UCM-MSCs show also a comparable positive coloration to Alcian Blue, however the latter being localized at intracellular level.

### Chondrogenic differentiation treatment induces a differential mRNA expression profile in UCM and UCB-MSCs

**Figure 2:**
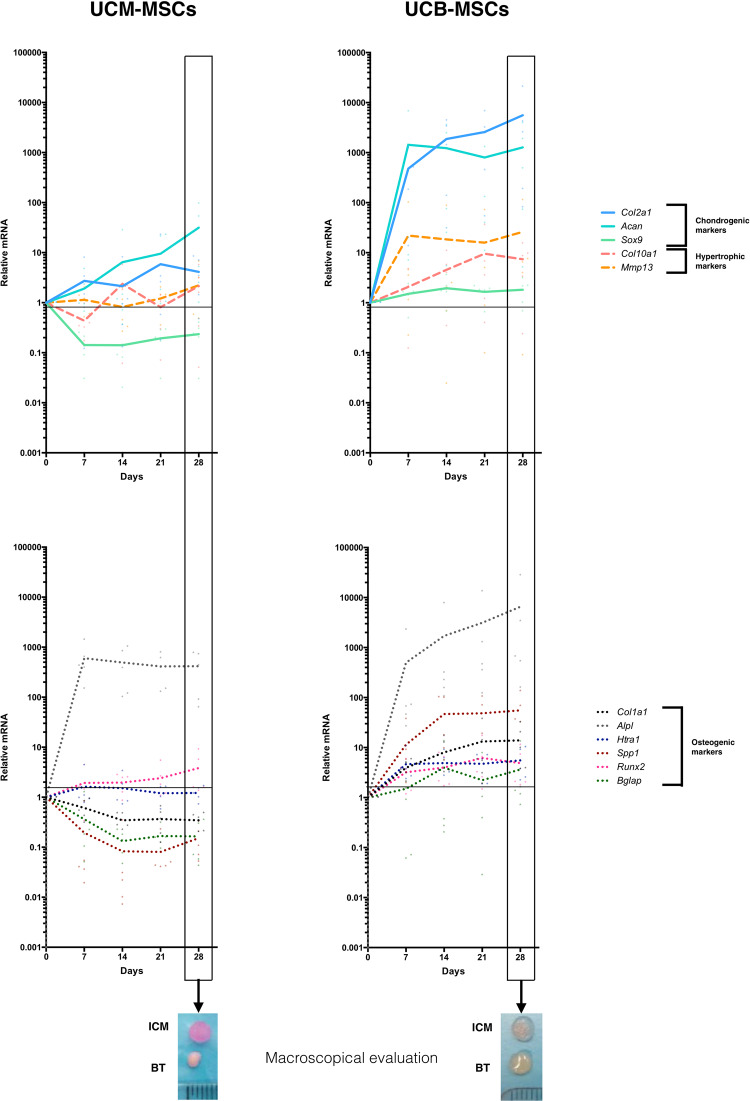
Strong differences between UCM-MSCs and UCB-MSCs in gene expression during chondrogenesis. Chondrogenesis was induced in UCM-MSCs and UCB-MSCs by culturing the cells in 3D scaffolds under BMP-2 and TGF-ß1 treatment. The time-course monitoring of chondrogenic (*Col2a1*, *Acan*, *Sox9*), hypertrophic (*Mmp13*, *Col10a1*) (upper panel) and osteogenic markers (*Col1a1*, *Alpl*, *Htra1*, *Spp1*, *Runx2* and *Bglap*) (lower panel) were assessed at 7, 14, 21 and 28 days post-treatment. At each time point, the mRNA levels of each transcript were normalized to the mRNA levels of the housekeeping gene. Relative expression of each gene was compared to the expression of the control (D0) set at 1. Small dots represent the distribution of the values measured for the 5 independent samples at each time. The images below the graphs represent the macroscopical appearance of the neo-tissue substitute following 28 days under the chondrogenic regimen (BT treatment).

The potential of UCM- and UCB-MSCs to be used in cartilage tissue engineering was further evaluated by seeding them in type I/III collagen scaffolds in a culture medium containing BMP-2 (50 ng/ml) and TGF-ß1 (10 ng/ml) (referred as BT condition here after). A time course analysis of the expression of 11 genes encoding chondrogenic, osteogenic and hypertrophic markers was performed by RT-qPCR at 7, 14, 21 and 28 days. Major differences in expression profile following stimulation with the chondrogenic medium were observed between UCM- and UCB-MSCs (Fig. 2). While the expression of the chondrogenic markers *Col2a1*, *Acan* and *Sox9* increased during the differentiation protocol, only few inductions were observed in UCM-MSCs. These results implicate a strong difference in cellular responses of both neonatal MSCs sources confirmed by macrospocical analysis at 28 days of chondrogenic differentiation (Fig. 2). Here, BT condition has been compared to Incomplete chondrogenic medium condition which represents a control culture condition without the stimulation by growth factors (referred as ICM condition here after). Following 28 days of BT treatment, UCB-MSCs produced a hydrated, nacreous tissue resembling cartilage, while UCM-MSCs showed a contracted appearance.

### Strong expression of cartilage-specific markers after chondrogenic differentiation for UCB-MSCs, but not for UCM-MSCs

**Figure 3:**
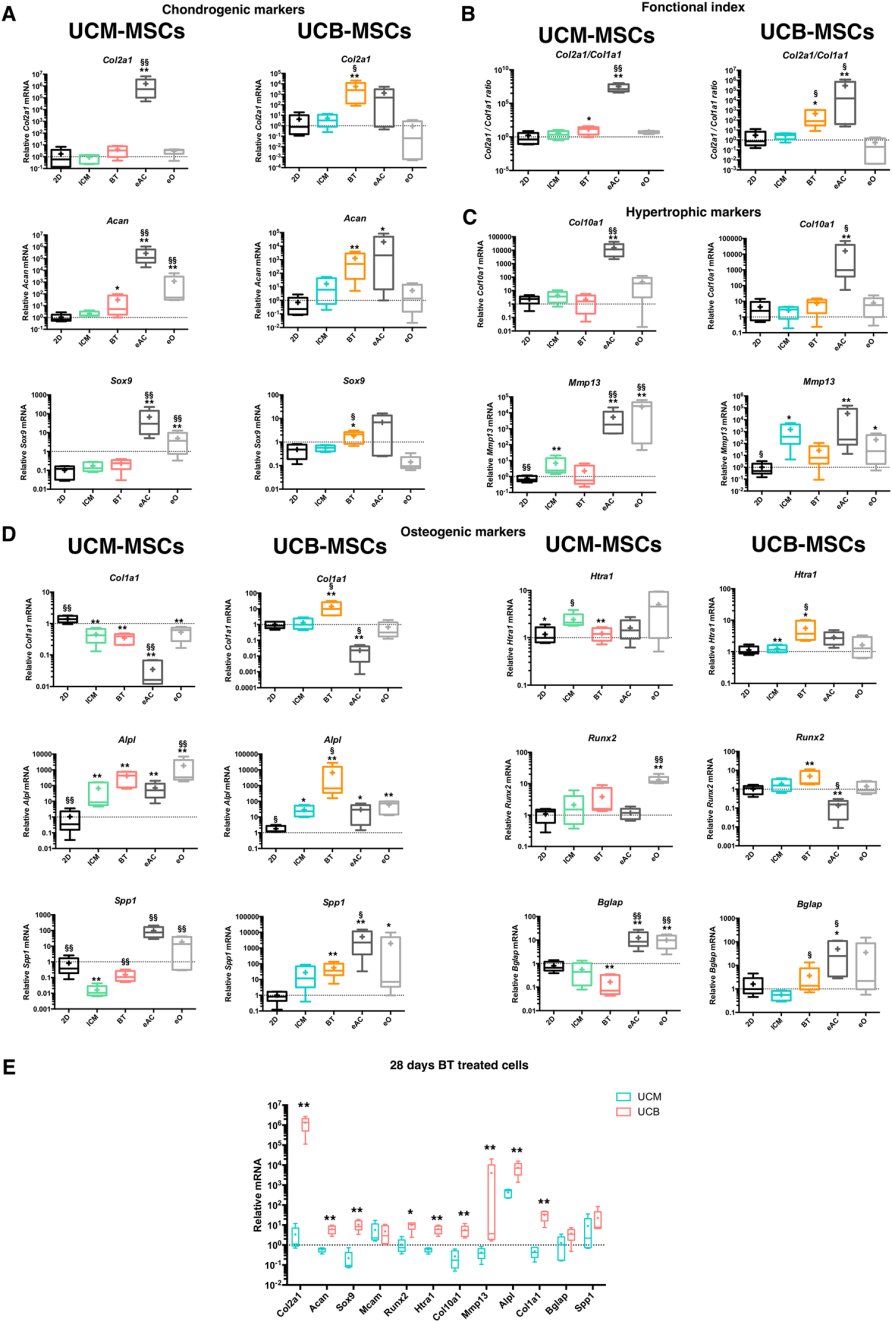
Differential expression of chondrogenic and osteogenic markers for UCM-MSCs and UCB-MSCs after chondrogenic differentiation in type I/III collagen scaffolds. UCM-MSCs and UCB-MSCs were cultured in type I/III collagen scaffolds and normoxia for 28 days in incomplete chondrogenic medium (ICM) or ICM supplemented with BMP-2 (50 ng/ml) and TGF-ß1 (10 ng/ml) (BT). Relative mRNA amounts encoding chondrogenic markers (**A**) (*Col2a1*, *Acan* and *Sox9*), hypertrophic markers (**C**) (*Col10a1* and *Mmp13*) and osteogenic markers (**D**) (*Col1a1*, *Alpl*, *Spp1*, *Htra1*, *Runx2* and *Bglap*) were determined by RTqPCR. The functional *Col2a1/Col1a1* ratio was presented in (**B**) mRNA extracts obtained from equine articular chondrocytes (eAC) released from cartilage after overnight enzymatic digestion and equine P0 osteoblasts (eO) were used as controls. Monolayer cultured MSCs (2D) were used as undifferentiated cells control. All results for each MSCs source were normalized versus untreated MSCs incubated during 1 h in type I/III collagen scaffolds at 37 °C and presented as the relative expression of each gene. Box-plots represent five independent experiments for each MSCs source. Statistically significant differences were determined using the Mann-Whitney test. *: 2D versus other conditions (*p < 0.05, **p < 0.01). §: ICM versus other conditions (^§^p < 0.05, ^§§^p < 0.01). (**E**) Steady-state levels of mRNA expression in UCB-MSCs and UCM-MSCs after 28 days of chondrogenic differentiation in BT condition were compared by RTqPCR. All results were normalized versus undifferentiated sample of one UCM-MSCs sample, and presented as the relative expression of each gene. Box-plots represent five independent experiments and statistically significant differences were determined using the Mann Whitney test by comparison of UCM-MSCs versus UCB-MSCs *:(*p < 0.05, **p < 0.01).

To evaluate the chondrogenic potential of both MSCs sources, we focused on the gene expression at the end of chondrogenic differentiation, at 28 days compared to undifferentiated cells cultured in monolayer (2D). Quality of the *de novo* synthesized ECM was evaluated by molecular analyses, using healthy equine chondrocytes as reference. Analysis of the chondrogenic markers expression after BT protocol showed large differences between the two MSCs populations. UCB-MSCs displayed strong expression of the chondrocyte markers *Col2a1*, *Acan* and *Sox9* compared to monolayer basal condition (2D) (1043-fold, 72-fold and 3-fold induction, respectively). Interestingly, mRNA levels of these chondrogenic markers were similar in differentiated UCB-MSCs and in primary culture of equine chondrocytes. By contrast, only a moderate induction of *Acan* expression was observed in UCM-MSCs following treatment (13-fold induction) whereas *Col2a1* and *Sox9* expressions were not modulated by treatments compared to 2D condition (Fig. 3A).

The functional *Col2a1/Col1a1* ratio was increased in both types of neonatal MSCs following differentiation, respectively 133-fold for UCB-MSCs and 9-fold for UCM-MSCs (Fig. 3B).

Chondrocyte hypertrophic markers expression was also evaluated following the differentiation process. Results showed that *Mmp13* mRNA expression is upregulated only for ICM condition in both MSCs sources in comparison to the 2D condition (9-fold for UCM-MSCs and 1429-fold for UCB-MSCs). *Col10a1* mRNA levels were unaffected by the experimental conditions and remained lower than *Col10a1* mRNA relative levels in eAC (Fig. 3C). High *Col10a1* mRNA steady-state amounts after post-enzymatic isolation of P0 eAC could be explained by the presence of hypertrophic chondrocytes in the sample during cartilage biopsy and chondrocytes isolation. However, as previously described, *Col10a1* mRNA decreases progressively after few cell passages probably because of the instability/death of the hypertrophic phenotype in monolayer cell culture (Rakic *et al*.^33^).

We paid a particular attention to the expression of osteogenic markers, to evaluate if our differentiation process may induce MSCs into another lineage (Fig. 3D). Results showed an increase of mRNA levels for *Alpl* in UCM-MSCs and UCB-MSCs (6-fold and 205-fold induction, respectively), while opposite results were observed for other markers. *Col1a1*, *Spp1, ß-Glap* and *Htra1* were downregulated in UCM-MSCs (respectively by 4-, 5-, 5- and 1-fold) and *Col1a1*, *Spp1* and *Htra1* were upregulated in UCB-MSCs (respectively by 16-, 63- and 5-fold). For Runx2, this transcriptional factor implicated in hypertrophy and osteogenesis, BT treatment induce its transcription only in UCB-MSCs compared to 2D condition (by 2-fold).

Finally, a comparative analysis of relative mRNA levels after 28 days of chondrogenic differentiation confirmed the stronger expression of chondrogenic markers in UCB-MSCs than in UCM-MSCs, from a quantitative point of view (Fig. 3E). After 28 days of chondrogenesis, comparative analysis between UCM-MSCs and UCB-MSCs shows a significant overexpression of the chondrogenic markers in UCB-MSCs in comparison to UCM-MSCs (respectively 3 × 10^5^-, 11- and 44-fold more for *Col2a1*, *Acan* and *Sox9*). Here, *Mcam*, encoding the CD146 surface marker and considered as a multipotency predictor was also quantified. No significant difference was observed in the *Mcam* expression at the end of differentiation between UCM- and UCB-MSCs. Interestingly, the mRNA levels of the osteogenic markers (*Bglap* and *Spp1*), deregulated during UCM-MSCs differentiation, were expressed at comparable level in UCM- and UCB-MSCs at 28 days of BT treatment. This last result implicates that osteogenic markers could be highly expressed at undifferentiated state in UCM-MSCs compared to UCB-MSCs.

**Figure 4:**
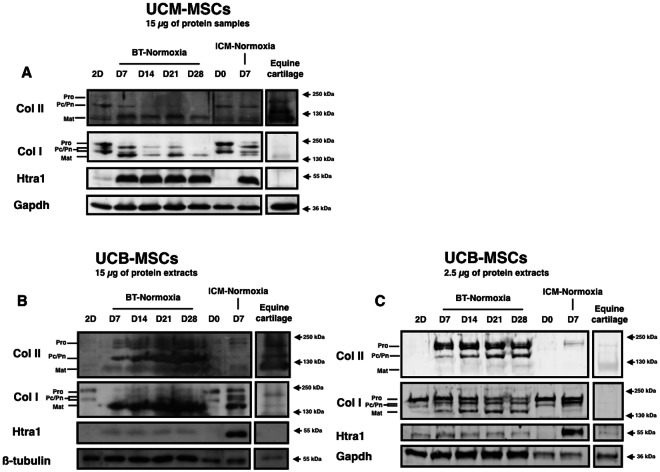
Protein analysis of UCM-MSCs and UCB-MSCs after chondrogenic differentiation. UCM-MSCs and UCB-MSCs were cultured in type I/III collagen scaffolds in normoxia during 7, 14, 21 and 28 days in ICM supplemented with BMP-2 (50 ng/ml) and TGF-ß1 (10 ng/ml). Protein extracts were analyzed in western-blots for type II collagen, type I collagen and Htra1. Representative blots of UCM-MSCs (n = 5) (**A**) and UCB-MSCs (n = 3) (**B** and **C**) are shown. (**C**) 2.5 µg of protein extracts were used for the western-blot of UCB-MSCs samples in order to enhance signal accuracy. The blots presented come from 2 independent experiments and the figure has been composed from 3 western-blots, each coming from one gel. Different levels of type II and type I collagen maturation forms are indicated such as type II procollagen (pro), without C- or N- terminal propeptides (Pc/Pn) and the mature doubly cleaved form (mat).

Western blots were performed to evaluate the protein levels for some of these markers and results were compared to healthy equine articular cartilage samples (Fig. 4). Despite the observed lack of increase in relative levels of *Col2a1* mRNA in response to BT treatment in UCM-MSCs, the mature type II collagen protein was detected after seven days of incubation (Fig. 4A). However, no further increase in the levels of this protein was observed in subsequent days, right up until the end of BT stimulation, after 28 days of culture. UCM-MSCs synthesized an immature form of type I collagen. BT treatment induced the maturation of this form, but the global level of type I collagen seems to decrease during the 28 days of cell culture both in ICM and BT conditions (Supp. Figure 1). High levels of Htra1, which is involved in matrix mineralization^34^, were induced by 3D cell culture with or without BT treatment, throughout chondrogenic differentiation.

High levels of ECM synthesis were induced by treatment of UCB-MSCs with chondrogenic factors (Fig. 4B). This induction made it possible for use to load much smaller amounts of protein extract onto SDS-PAGE gels (once sixth the amount) to evaluate more correctly signal differences in the intensity (Fig. 4C). The use of less than 2.5 µg UCB-MSCs protein for SDS-PAGE led to a loss of the peroxidase signal for some of the antibodies used in the analysis, in particular for reference proteins. Type II procollagen (Pro) was found to be overexpressed after chondrocyte differentiation, on gels loaded with 2.5 µg protein. Type II procollagen with C-terminal or N-terminal propeptide deletion (the Pc and Pn forms, respectively) was also produced by UCB-MSCs treated with BT. Nevertheless, low levels of the mature form of type II collagen were detected. In parallel, high levels of type I collagen were observed. BT treatment induced the maturation of this collagen isotype, as the amount of the procollagen form seemed to decrease with treatment time, suggesting a likely blockade of type I collagen synthesis. By contrast, Htra1 expression, which was induced by 3D culture, was inhibited by BT treatment in UCB-MSCs, but not in UCM-MSCs. Chondrogenic medium induced higher levels of ECM production in UCB-MSCs than in UCM-MSCs, as shown by SDS-PAGE for equal amounts of protein. For both MSCs sources, no type X collagen has been detected (Supp. Figure 2).

### UCB-MSCs express a hyaline-like cartilage ECM under BT treatment, whereas UCM-MSCs do not

**Figure 5:**
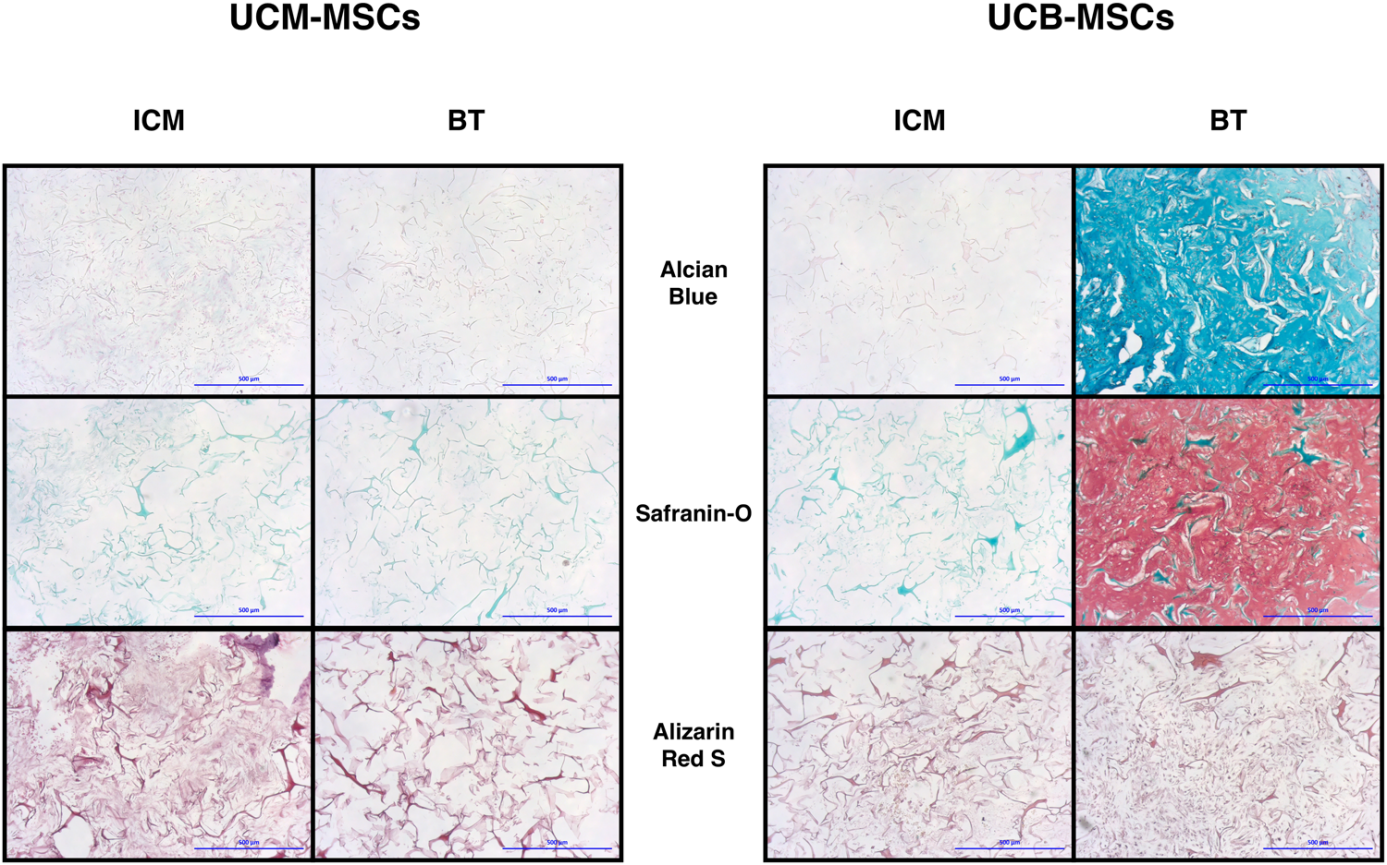
Histological analysis of the neo-tissue substitute generated by UCM-MSCs and UCB-MSCs after chondrogenic stimulation. UCM-MSCs and UCB-MSCs were cultured in type I/III collagen scaffolds at 21% O_2_ during 28 days in ICM supplemented with or without BMP-2 (50 ng/ml) and TGF-ß1 (10 ng/ml). Chondrogenic and/or osteogenic ECM was analyzed by alcian blue, O-safranin and alizarine red S stainings. A representative example from four samples for UCM-MSCs and three samples for UCB-MSCs is shown (magnification: ×10, scale bar: 500 µm).

Following treatment under chondrogenic, osteogenic and adipogenic differentiation protocols, tissue substitutes were processed for histological evaluations. Differentiated UCB-MSCs showed intense Alcian blue and O-safranin labeling, while a moderate staining was observed for UCM-MSCs. In some UCM-MSCs substitutes, ECM mineralization was observed, as assessed by Alzarin red staining (Fig. 5).

### UCB-MSCs and UCM-MSCs differ not only in chondrogenic properties, but also in intrinsic characteristics

**Figure 6:**
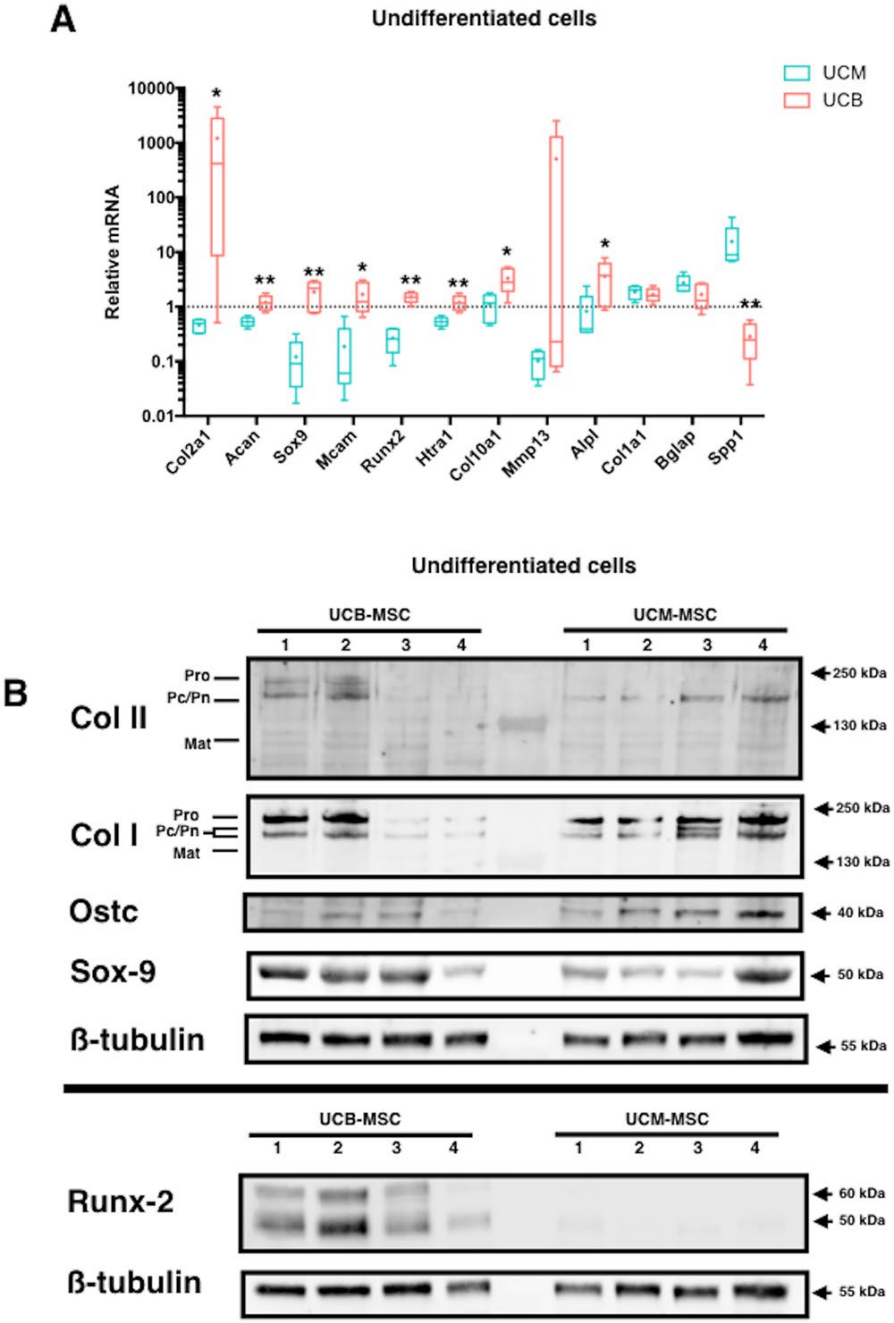
Comparison of the osteo-chondrogenic markers expression between UCM-MSCs and UCB-MSCs in longitudinal study. UCM-MSCs and UCB-MSCs were cultured in monolayer to sub-confluence. Steady-state levels of mRNA expression in UCB-MSCs and UCM-MSCs were compared by RTqPCR. All results were normalized versus undifferentiated sample of one UCM-MSCs sample, and presented as the relative expression of each gene. Box-plots represent five independent experiments and statistically significant differences were determined using the Mann Whitney test by comparison of UCM-MSCs versus UCB-MSCs *: *p < 0.05, **p < 0.01. (**B**) Four samples of monolayer cultured MSCs for each MSCs sources (numbers 1 to 4) were compared at protein level by western-blot analysis of types I and II collagens, osteocacin, Sox-9 and Runx-2. The images presented correspond to a western-blot including 2 gels.

Based on the previous results showing contrasting results of UCM- and UCB-MSCs in response to chondrogenic stimulation, a more accurate evaluation of both MSCs populations in steady-state conditions was carried out (Fig. 6). Results showed significant differences between both cell types at basal level (Fig. 6A). UCB-MSCs express higher levels of chondrogenic markers than UCM-MSCs (respectively 2500-, 2- and 15-fold more for *Col2a1*, *Acan* and *Sox9*), while the osteogenic marker *Spp1* was significantly more expressed in UCM- than UCB-MSCs (55-fold difference) (no difference in gene expression was observed for *Alpl Col1a1* and *Bglap*). *Mcam*, was also more strongly expressed in UCB- than in UCM-MSCs (8-fold difference).

At the protein level, in a longitudinal study of 8 different samples of neonatal populations (Fig. 6B), basal level of type II collagen seems to be higher in UCB- than UCM-MSCs. Higher expression of type I collagen was found in UCM-MSCs compared to UCB-MSCs. Interestingly, osteocalcin protein levels were much higher in UCM-MSCs than in UCB-MSCs. Another major difference between the MSCs from these two different sources was the overexpression of the osteochondral transcription factors Sox-9 and Runx-2 in UCB-MSCs compared to UCM-MSCs.

### Hypoxia induces a time-dependent effect on transcript expression profile in UCM- and UCB-MSCs during chondrogenesis induction

**Figure 7:**
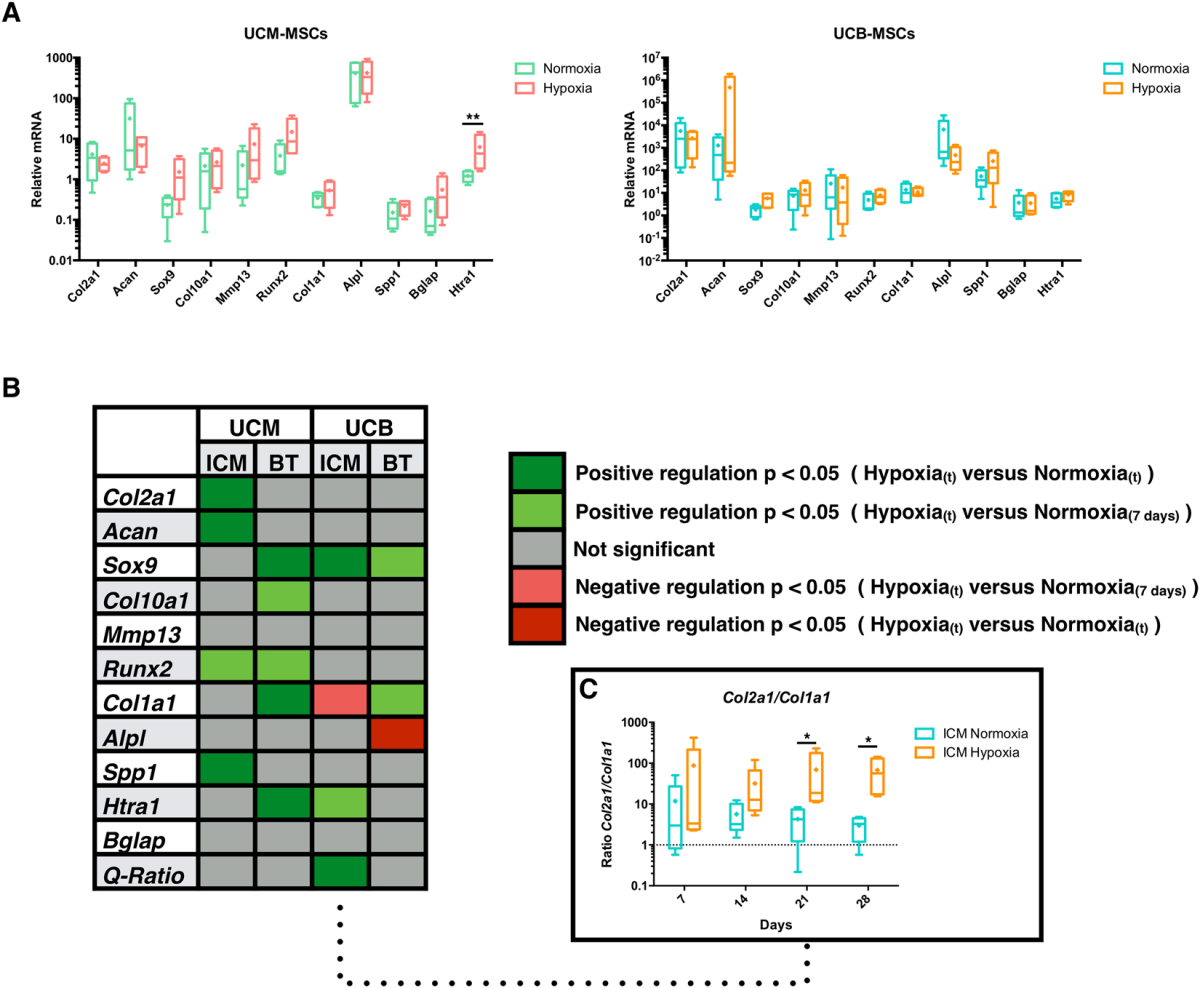
Differential gene expression modulation under hypoxia during a kinetic chondrogenic differentiation of UCM-MSCs and UCB-MSCs. UCM-MSCs and UCB-MSCs were cultured in type I/III collagen scaffolds at 21% or 3–5% O_2_ during 7, 14, 21 and 28 days in ICM supplemented with BMP-2 (50 ng/ml) and TGF-ß1 (10 ng/ml). Relative mRNA expression was determined by RTqPCR. All results for each MSCs source were normalized versus untreated MSCs incubated during 1 h in type I/III collagen scaffolds at 37 °C and presented as the relative expression of each gene. (**A**) Effect of hypoxia on chondrogenic differentiation after 28 days of culture in the presence of growth factors. (**B**) Summary of the relative effects of hypoxia on different genes expression: differential time-dependent profile between MSCs sources and upon growth factors treatment. Manual classification of mRNA expression variation by hypoxia was realized by taking into account only the significant results between hypoxia versus normoxia at the same time of measurement or between hypoxia versus normoxia at 7 days. (**C**) *Col2a1/Col1a1* ratio (Q-Ratio) was presented for UCB-MSCs cultured in ICM. Box-plots represent five independent experiments for each MSCs source. Statistically significant differences between normoxia and hypoxia conditions were determined using the Mann Whitney test. *: Hypoxia versus normoxia at the same time (*p < 0.05, **p < 0.01).

Hypoxia is known to affect positively the chondrogenic differentiation of BM-MSCs *in vitro*. This parameter has not yet been evaluated in equine UCB- and UCM-MSCs. Gene expression of chondrogenic markers has been compared in UCB- and UCM-MSCs cultured in BT condition under normoxia and hypoxia for 28 days. Molecular analysis showed that hypoxia did not induce significant difference in gene expressions for the selected genes (Fig. 7). Only *Htra1* showed a significant modulation of expression in UCM-MSCs under hypoxia (5-fold increase in hypoxia).

To evaluate if hypoxia may nonetheless exert some effect throughout the chondrogenesis process, a time-course gene expression analysis was realized. Results showed that hypoxia may induce various effects between UCM- and UCB-MSCs throughout the differentiation process, but not conclusive (Fig. 7B and Supp. Figures 3, 4). These data are summarized in Fig. 7B, in which we classified the significant results for comparisons between hypoxia and normoxia at the same time points *[Positive or negative regulation p* < *0.05* (*Hypoxia*(*t*) *versus Normoxia*(*t*))*]* (strong effect) and between hypoxia at the end of the study and seven days of normoxia *[Positive regulation p* < *0.05* (*Hypoxia*(*t*) *versus Normoxia*(*7 days*))*]* (weaker effect). During our 3D cell culture model, UCM-MSCs and UCB-MSCs responded differently to hypoxia, in ICM or BT conditions. These differences were gene- and time-dependent, with opposite results between UCB- and UCM-MSCs in some cases.

Hypoxia seems to have also an implication in chondrogenic differentiation without the use of growth factors (Fig. 7B,C and Supp. Fig. 4). At the end of cell culture, hypoxia condition induced an increase of *Sox9* expression and *Col2a1/Col1a1* ratio (respectively 2-fold and 21-fold for *Sox9* and *Col2a1/Col1a1* ratio at 28 days in hypoxia condition compared to 28 days in normoxia condition).

Hypoxia seems also able to repress *Alpl* expression during UCB-MSCs chondrogenesis (57-fold decreased in hypoxia condition compared to normoxia condition at 14 days) (Supp. Fig. 3).

### Hypoxia decreases the amount of hyaline-cartilage ECM synthesized by UCB-MSCs

**Figure 8:**
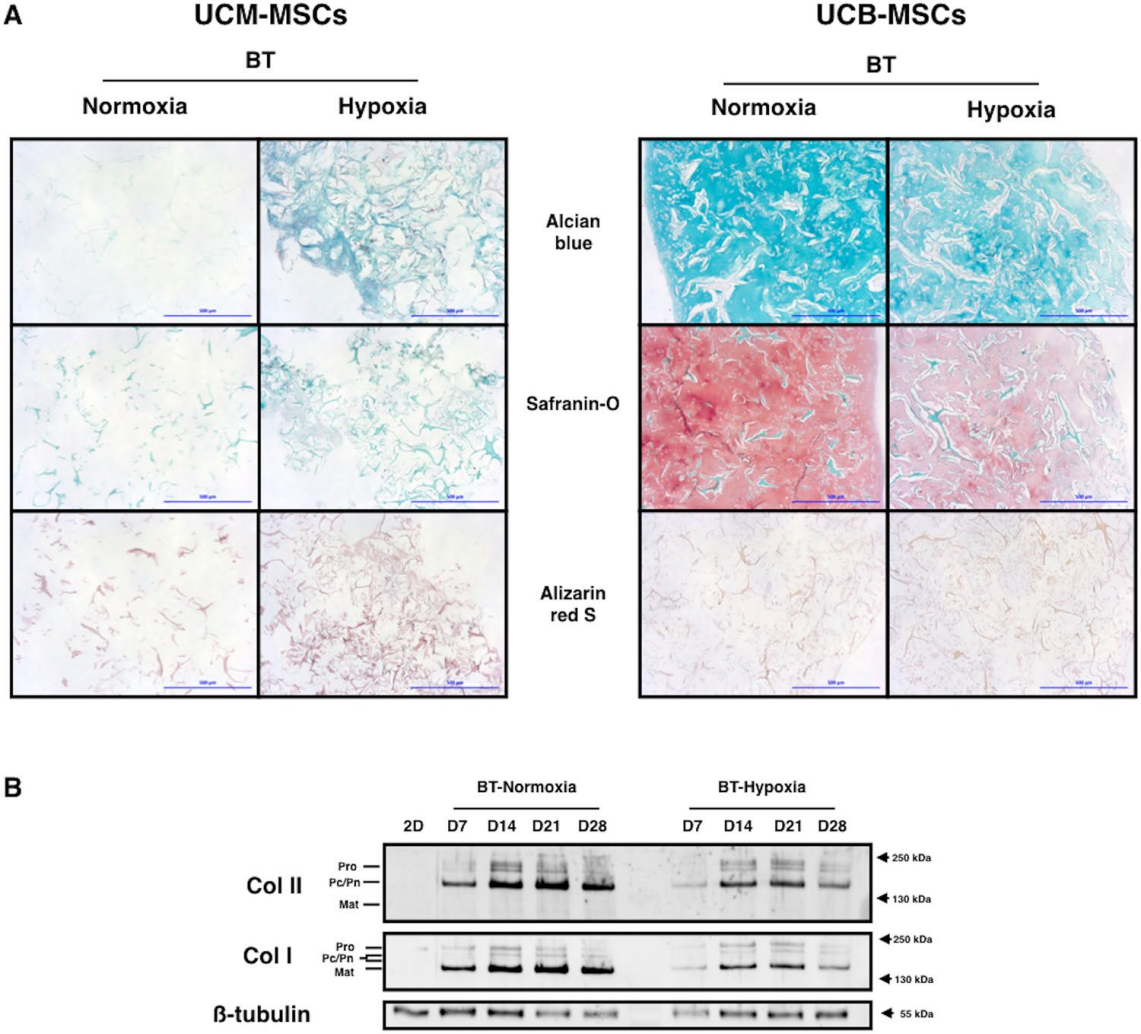
Hypoxia influence on the nature of the matrix newly synthesized by UCB-MSCs under BT treatment. UCB-MSCs were cultured in type I/III collagen scaffolds at 21% or 3–5% O_2_ during 7, 14, 21 and 28 days in ICM supplemented with BMP-2 (50 ng/ml) and TGF-ß1 (10 ng/ml). (**A**) The qualitative nature of the ECM was evaluated by alcian blue, O-safranin and alizarine red S stainings. A representative example from four samples for UCM-MSCs and three samples for UCB-MSCs are shown (magnification: ×10, scale bar: 500 µm). (**B**) Protein extracts were analyzed in western-blots for type II and type I collagens. Representative blots of UCB-MSMs (n = 3) are shown. The blots come from one experiment and a western-blot from 1 gel. 2.5 µg of protein extracts were used for SDS-PAGE of UCB-MSCs samples to improve signal accuracy. Different levels of type II and type I collagen maturation forms are indicated such as type II procollagen (pro), with only C- or N- terminal propeptides (Pc/Pn) and the mature doubly cleaved form (mat).

A histological analysis of the newly synthesized ECM produced by neonatal MSCs was performed after chondrogenic differentiation in different oxygen concentrations (Fig. 8). Oxygenation conditions had only minor effects on the new tissue synthesized from UCM-MSCs. They synthesized a highly diffuse ECM with a low glycosaminoglycan content and low levels of matrix mineralization under BT treatment in normoxia. The synthesis of this ECM was further reduced by incubating the cells in hypoxia.

Hypoxia affected all samples of UCB-MSCs subjected to histological analysis. It seemed to decrease ECM synthesis and resulted in a more diffuse and heterogeneous ECM than observed in BT-normoxia conditions. As ECM was already poorly mineralized in normoxia, it was difficult to detect an effect of hypoxia on calcium deposits, although such an effect was observed in some cases (Fig. 8A).

These results were confirmed by western-blot analyses of collagen levels. Total collagen synthesis was decreased by hypoxia during the differentiation of UCB-MSCs (Fig. 8B). Similar decreases were observed for both type I and type II collagen. Thus, the lower levels of ECM synthesis in hypoxia were not associated with a change in ECM quality. UCM-MSCs synthesized only very small amounts of ECM under BT conditions; we did not highlight an effect of hypoxia at protein level for this neonatal MSCs source (Supp. Fig. 5).

## Discussion

There are great hopes for the use of MSCs in regenerative medicine and, particularly, in tissue engineering, due to their cellular plasticity and numerous biological properties. In the engineering of both horse and human cartilage tissues^35^, MSCs may supplant chondrocytes for matrix-induced autologous chondrocyte implantation (MACI), thereby overcoming the medical and cellular limitations associated with the need for cartilage biopsy and chondrocyte amplification^36^. In particular, neonatal MSCs appear as a valuable alternative to develop cell- and tissue-based products. Clarification of their biological properties is required before their therapeutic use can be considered. In this study, we aimed at evaluating the properties of 2 neonatal tissues-derived MSCs from horse, based on conventional characterization (ISCT recommendations). Moreover, we investigated the chondrogenic differentiation potential of both UCM- and UCB-MSCs for their use in tissue engineering.

While equine UCM- and UCB-MSCs share the common characteristics usually reported for MSCs, we were able to maintain UCM-MSCs proliferation in culture for longer period than UCB-MSCs. Most of UCB-MSCs samples stop proliferating after 4 passages. These results reveal an heterogeneity among UCB-MSCs strains, as only 4 of the samples have been maintained for at least 4 passages and 3 for 5 passages in culture. Only one UCB-MSCs sample has been grown up to 13 passages (data not shown), while UCM-MSCs could be reproducibly maintained up to 13 passages. This difference of proliferative profile could be explained either by intrinsic differences among the biological sources, or by different cell responses for the same culture process, potentially better adapted to UCM-MSCs. Higher batch to batch consistency with UCM-MSCs suggest that these cells might be more resistant to the stress induced by shipping and/or freeze/thaw steps. Nevertheless, their heterogeneity and the difficulties to maintain UCB-MSCs in cell culture does not seem to affect their much higher chondrogenic potential.

When considering equine UCM- and UCB-MSCs for their specific use in cartilage tissue regeneration, major differences were observed between both cell populations. Following the differentiation process, molecular analysis of UCB-MSCs showed strong expression of chondrogenic markers (*Col2a1, Acan, Sox9*), while these genes were poorly induced in UCM-MSCs. Furthermore, histological analysis showed that UCB-MSCs produced an abundant ECM composed of type II collagen and sulfated GAGs, while UCM-MSCs express only a limited amount of ECM. To rule out the possibility that our culture conditions or 3D collagen scaffold model was not suitable to induce chondrogenic differentiation of UCM-MSCs, conventional methods (including micro-pellet and hanging drop models)^37^ were further evaluated. None of the different chondrogenic conditions tested promotes substantial cartilage-specific markers expressions by UCM-MSCs (Supplementary Figs S6–S8).

If we focus on the UCB-MSCs chondrogenesis, no mineralization has been observed in the neo-cartilage and type X collagen transcript and protein seem to be expressed at very low amounts during this differentiation in normoxia. Nevertheless, overexpression of Runx2, Spp1, Alpl and type I collagen seems to indicate an hypertrophy of MSCs and a possible osteochondral ossification. The weak expression of Htra1 protein during UCB-MSCs chondrogenesis seems to corroborate these results even if no induction of Mmp13 transcription was observed. Indeed, in addition to Mmp-13 and Mmp-10 up-regulation, cartilage hypertrophy events could induce a deep remodeling of the ECM by protease expression and activation that may lead to the formation of bone collar^38^. Then, Mmp-9 and Mmp-14 will ensure the removing of the bone collar which is replaced by a bone specific ECM.

Recently, HTRA1 was described as a catabolic and transition actor of hypertrophy and ECM remodeling. Its expression and activity can regulate Mmp-13 expression, hypertrophic commitment and the mineralization of the early bone ECM^39–41^. Strategies targeting Htra1 activity or expression could be relevant. Indeed, this serine protease could be involved in the MSCs lineages and could play a key role in the chondrogenesis and the MSCs behavior^33,42,43^. To better characterize the differentiation status of UCB-MSCs, protease activity and/or zymographic analyses and/or protease inhibitor assays need therefore to be considered in future experiments.

Hypertrophic cartilage represents a transition tissue at the frontier of an hydrated/viscoelastic tissue and a calcified/mineralized cartilage matrix. During embryonic and post-natal development, hypertrophic chondrocytes participate to remove the limb cartilage pattern and assist the endochondral bone formation^28^. Concerning articular cartilage tissue engineering, if cartilage hypertrophy happens, this will lead to an imperfect neo-cartilage substitute which can lose its biomechanical properties and generates an instable tissue after implantation. Indeed, chondrocyte hypertrophy can also promote cell apoptosis and decellularization of the tissue with the association of reactive oxidative species release. Furthermore, an hypertrophic tissue could provide a place for vascularization which impairs the general integrity of the implanted articular cartilage^44^.

The limitation of the hypertrophic commitment in the mature chondrocyte represents a real challenge and, in this case, the control of the proximal microenvironment is a critical parameter to stabilize the articular chondrocyte phenotype. Terminal differentiation of the chondrocyte is mediated by several molecular actors and *Runx2* is a major one^45^. By contrast to the normal chondrogenic differentiation, its expression was up-regulated in UCB-MSCs in our experiments. One way to inhibit Runx2 could be the activation of the hypoxia inducible factor-1 alpha (HIF-1α). Whereas the 3–5% O_2_ hypoxic culture inhibits *Alpl* transcription during UCB-MSCs chondrogenesis in our experiments, it is not enough to repress *Runx2* expression. This Runx-2 repression could be obtained by lowering the oxygen concentrations near to 1% or by cobalt chloride treatment which is able to induce a Runx-2 *trans*inhibition by an effective HIF-1α activation^46^. Another solution to inhibit this terminal differentiation of the chondrocytes could be to increase the cell density for the chondrogenic commitment^47^.

Molecular analysis of chondrogenic and osteogenic markers in undifferentiated UCM- and UCB-MSCs showed that these cells have different molecular expression patterns in basal condition. UCM-MSCs express higher levels of the osteogenic markers *Spp1* (the gene encoding the osteopontin protein) and osteocalcin which suggest a possible early osteogenic commitment. UCB-MSCs, on the other hand, significantly overexpress the osteochondral transcription factors Sox-9 and Runx-2. This could be potentially associated with their greater tendency to differentiate into mature cells of the osteochondrogenic lineage^45^. Interestingly, *Mcam*, encoding the melanoma cell adhesion molecule (CD-146) was only expressed in UCB-MSCs. This marker has been described as a predictive multipotency marker of particular relevance in chondrogenic differentiation^48,49^. Altogether, these data suggest that UCB-MSCs are able to differentiate toward the chondrogenic lineage, while UCM-MSCs chondrogenesis seems to be limited. Interestingly, low expression of mature type II collagen protein was observed in UCM-MSCs after 7 days of differentiation. This chondrogenic marker expression could be due to a small population of chondroprogenitors within UCM-MSCs, which is not enough to synthesize significant amount of hyaline ECM. In fact, we supposed that differences observed between UCM-MSCs and UCB-MSCs could be caused by different proportions of cell progenitors in the native MSCs populations. Deep analysis of this intra-population heterogeneity could provide a better characterization of the different MSCs sources and their more efficient use in clinical applications^50^.

Overall, our results showing a limited commitment of UCM-MSCs towards the chondrogenic lineage confirm the findings of Islam *et al*.^51^ reporting a poor chondrogenesis of UCM-MSCs, when using a multiple quantitative approach for measuring genes and proteins. These results are nonetheless in contradiction with other groups reporting the use of UCM as a suitable source for cartilage tissue engineering^24–26,52–54^. Divergent outcomes between groups may be due to many factors, in particular to the isolation and handling techniques of MSCs, the culture methods, and the treatments used to induce chondrogenesis.

A plausible explanation could be also that umbilical cord includes an heterogenous composition of MSCs which could drastically affect the nature of MSCs populations isolated from the whole tissue. This reasoning can be corroborated by the study of Sarugaser *et al*.^55^, who observed a high heterogeneity in the location of CD146+ cells in the umbilical cord structure. These cells called Human Umbilical Cord PeriVascular (HUCPV) cells are predominantly localized close to the vascular system of the umbilical cord. These studies and our work seem to indicate that the cord matrix should not be considered as a whole for clinical application but as an heterogenous tissue.

Hypoxia plays a major role in cartilage and chondrocyte physiology. We previously showed that hypoxia enhances chondrogenesis and hyaline-like ECM synthesis in human BM-MSCs^42^. Hypoxia is involved in the inhibition of the terminal differentiation of chondrocytes^31^, and in MSCs physiology as it is a key microenvironmental parameter^56,57^. Our results showing an inhibition of *Alpl* in UCB-MSCs during chondrogenesis in hypoxia corroborate these previous data. Nevertheless, while hypoxia did not induce a significant difference in gene expression of chondrogenic markers in differentiated UCB-MSCs, it reduces ECM synthesis. Only *Sox9* expression is overexpressed under hypoxia. This can be explained by the affinity of Hif-1α to consensus sequences in the promoter of the *Sox9* gene. This relation between Sox-9 and hypoxia is essential to the pre-chondrogenic induction under low oxygen conditions^58^.

Concerning our MSCs comparison, long-term hypoxia incubation during chondrogenesis of both types of neonatal MSCs induces variable cellular responses and confirm the differential regulation between both the MSCs sources. Hypoxia predominates in hematopoietic stem cell niches^59^ and may regulate the therapeutic properties of MSCs^60^. However, hypoxia has not clearly been involved in chondrogenesis of neonatal MSCs. Indeed, unlike bone marrow niche that maintains stem cells in a quiescent state through an hypoxic environment, MSCs in the cord blood evolve in a normoxic environment. For example, in the arterial umbilical cord blood, oxygen saturation is near to 99%^61^. Conversely, oxygen distribution is diffused across a gradient according to the proximity of the vessels in the UCM. This difference of *in vivo* microenvironment for both neonatal MSCs could explain the variations observed during the chondrogenic assays under hypoxic condition.

As previously reported, 3D-chondrogenesis of UCB-MSCs is more efficient when cultures are first placed in normoxia^62^, while hypoxia allows chondrocyte phenotype stabilization, as confirmed in our work by a down-regulation of the osteogenic and hypertrophic factor Alpl under hypoxia. Hypoxia stress at the beginning of the chondrogenesis probably imposes metabolic changes on UCB-MSCs, limiting ECM synthesis. Hypoxia could have a time-dependent effect on UCB-MSCs lineage commitment and cellular behavior. The long-term culture of undifferentiated cells in hypoxia before their differentiation may result in cellular metabolism adaptation, with differential effects on UCB-MSCs chondrogenesis.

This work provides the first extensive evaluation of the chondrogenic potential of equine UCM- and UCB-MSCs. It is also the first study, to our knowledge, investigating the role of hypoxia in chondrogenesis for both neonatal MSCs populations. Our results suggest that UCB-MSCs should be preferred over UCM-MSCs for cartilage generation and highlight the need to take inter-source variability into account for tissue engineering for therapeutic purposes in both humans and horses. These results also suggest that the native environment of each type of MSCs should be considered to establish optimal experimental conditions for growing and differentiating MSCs.

## Materials and Methods

### Equine mesenchymal stem cell samples

Equine UCM-MSCs and UCB-MSCs were kindly provided by Vetbiobank Inc. Briefly, neonatal tissues were recovered from selected mare belonging to French National Studs (IFCE, Saumur, France) following parturition in accordance with standard veterinary practice. All procedures were carried out in accordance with the guidelines put in place by IFCE for animal welfare.

UC samples (n = 8) and UCB bags (n = 9) were transported under appropriate refrigerated condition to the laboratory facility into 72 h. Samples processing and cell isolation were performed according to Vetbiobank’s protocols. Cells were grown in a proprietary medium and harvested at passages 1–3. UCB- and UCM-MSCs were cryopreserved in liquid nitrogen until further use. A microbiological screening was performed on both the maternal blood and the neonatal tissues.

### Culture of equine UCM-MSCs and UCB-MSCs

MSCs vials were thawed at 37 °C, and cells were grown in proliferation medium for several passages, with media renewal twice a week. At each passage, cells were trypsinized and counted by trypan blue exclusion assay. Cell proliferation was evaluated using the following equation:

∆t: time between two passages (h); N0: initial number of cells; Nt: cell number at harvesting; n: number of cell generations during ∆t; k: number of generations by time units; g: doubling time (h). Doubling times (g) exceeding 400 h caused by the rapid loss of cell proliferation were systematically excluded from analysis and considered as non-proliferating cells.

$$k=\frac{n}{\Delta t}=\frac{\log \,{N}_{t}-\,\log \,{N}_{0}}{\log \,2\times \Delta t}$$

$$g=\frac{1}{k}$$

### UCB- and UCM-MSCs characterization

#### Flow cytometry

MSCs at P2 to P4 were harvested, washed and resuspended in DPBS-sodium azide buffer (0.1%) at a density of 1 × 10^6^ cells/ml. Cells were incubated with monoclonal antibodies, for 25 min at 4 °C in a dark room. Monoclonal antibodies against the following proteins were used: CD-90, CD-44, MHC-1, MHC-2, CD-45 and CD-29 (with mouse anti-human CD90-PE, mouse anti-human CD44-APC, mouse anti-human CD29-PE (all three purchased from BioLegend), mouse anti-horse MHC1-FITC, mouse anti-horse MHC2-FITC, and mouse anti-human CD45-FITC (all four purchased from ABD Serotec)). Negative control staining was performed with the corresponding conjugated isotype antibodies. The cells were washed and resuspended in 300 µl D-PBS. Flow cytometry was performed with a BD ACCURI C6 cytometer (BD Biosciences).

### Evaluation of MSCs multipotency

We evaluated the chondrogenic, osteogenic and adipogenic differentiation of MSCs from P4 to P6, by specific staining in six-well plates. Chondrogenesis was induced at 70% confluence. MSCs were cultured in differentiation medium consisting of incomplete chondrogenic medium (ICM: HG-DMEM Glutamax (Thermo Fisher), dexamethasone (1 × 10^−7^ M, Sigma-Aldrich), ascorbic acid-2-phosphate (50 µg/ml, Sigma-Aldrich), proline (40 µg/ml, Sigma-Aldrich), sodium pyruvate (1 nM, Thermo Fisher), insulin transferin selenium (1%, Thermo Fisher)) supplemented with BMP-2 (50 ng/ml, rhBMP-2, inductOs, Wyeth Europa Ltd) and TGF-ß1 (10 ng/ml, Miltenyi Biotec). The chondrogenic differentiation medium was changed twice weekly during the 21-day incubation period. Osteogenesis was induced at 70% confluence. MSCs were cultured in osteogenic medium consisting of MSCs amplification medium supplemented with dexamethasone (1 × 10^−7^ M), ascorbic acid-2-phosphate (100 ng/ml), ß-glycerophosphate (10 mM). The osteogenic medium was changed once weekly during the 21-day incubation period. Adipogenesis was induced at 50% confluence. MSCs were cultured in adipogenic medium consisting of LG-DMEM, 1% glutamine, 2% PSF supplemented with 10% rabbit serum (Sigma-Aldrich), dexamethasone (1 × 10^−6^ M, Sigma-Aldrich), 3-isobutyl-1-methyl-xanthine (0.5 mM), indomethacin (0.2 mM) and recombinant human insulin (10 µg/ml). The medium was replaced three times during the first two weeks and the incubation was prolonged for seven more days. After 17–21 days of incubation, lipid droplet accumulation became visible. For each differentiation, MSCs cultured in amplification medium were used as negative controls.

At the end of the incubation period, cells were washed twice with DPBS, fixed with 10% formalin neutral buffer (Sigma-Aldrich) for 10 min at room temperature and then washed again with DPBS. Chondrogenesis was evaluated by staining with 1% alcian blue 1% in 1 M chlorhydric acid 1 M (pH = 1) (Sigma-Aldrich). Osteogenesis was assessed by alizarin red S (pH = 4.1) staining (Sigma-Aldrich) and adipogenesis was evaluated by staining with 0.3% oil red O in isopropanol (Sigma-Aldrich). Images were acquired with a confocal phase-contrast microscope.

### Chondrogenic differentiation in a 3D scaffold

Collagen scaffolds were manufactured by Symatèse Biomatériaux (Chaponost, France) and used for chondrogenic differentiation. These collagen sponges (100% collagen, 2 mm thick, 5 mm in diameter, corresponding to a volume of 0.04 cm^3^, with a pore size of about 100 μm) consisted of native type I (90–95%) and type III (5–10%) collagens from calf skin. Collagen fibers were crosslinked with glutaraldehyde to increase their stability, sterilized by β-irradiation and they displayed no swelling on rehydration.

After MSCs amplification, P3-P6 cells were treated with trypsin, stained with trypan blue, counted and included in collagen scaffolds. The collagen sponges were seeded with cells by dropping 20 μl of the cell suspension onto the scaffold (5 × 10^5^ cells/scaffold) in 96-wells culture plates, drying for 1 h at 37 °C under an atmosphere containing 5% CO_2_ and then transferring the cells to ICM or ICM supplemented with 50 ng/ml BMP-2 and 10 ng/ml TGF-ß1 in 24-well plates. For cultures in hypoxic conditions, ICM was pre-equilibrated at 3% O_2_ by bubbling before the transfer of culture dishes into hypoxic Plas-Labs basic multistation glove box (Sigma-Aldrich) containing 2–4% O_2_ at 37 °C, under an atmosphere containing 5% CO_2_. MSCs were allowed to differentiate into chondrocytes for 7, 14, 21 or 28 days and were then harvested for RNA, protein and histochemical analyses. MSCs incubated for 1 h in collagen type I/III scaffolds at 37 °C were used as day 0 controls for these experiments.

### RNA analysis

Total RNA was extracted in Trizol Reagent (Thermofisher) according to the manufacturer’s instructions. We reverse-transcribed RNA to generate cDNA, using reverse transcriptase (MMLV, Thermo Fisher) and oligodT (Eurogentec). RT-qPCR was performed on a StepOnePlus Real-Time system (Thermo Fisher) by Power SYBR Green PCR (Thermo Fisher). The sequences of the primers used are listed in Supplementary Table S2. They were purchased from Eurogentec. The ß-actin gene (*Actb*) was used as an endogenous reference gene. Relative mRNA levels were determined with StepOnePlus software (Thermo Fisher), by the 2−∆∆CT method or the standard curve method, depending on the efficiency of amplification for *Actb* amplification and each target gene. The results are expressed as the mean for triplicate samples^63^.

### Protein extraction and western-blots

After treatment, the scaffolds containing the cells were rinsed once with ice-cold DPBS (PAN- Biotech), frozen, crushed and total protein was extracted in RIPA lysis buffer supplemented with protease inhibitors. Protein concentration was assessed with the Bradford colorimetric assay (Bio-Rad). We separated 2.5–15 µg of total protein by electrophoresis in 10% polyacrylamide gels (Bio-Rad) in denaturing conditions and transferred the proteins onto a polyvinylidene difluoride membrane (PVDF) (Biorad) with the Trans-Blot Turbo Transfer System (Biorad). Non-specific binding sites on the membrane were blocked by incubation with 10% non-fat milk powder in Tris-buffered saline supplemented with 0.1% Tween for 2 h. Membranes were incubated overnight at 4 °C with rabbit anti-human type I collagen (Novotec), rabbit anti-human type II collagen (Novotec), rabbit anti-human type X collagen (Abcam or Sigma-Aldrich), rabbit anti-human HtrA1 (Millipore), mouse anti-human Sox9 (Santa Cruz Biotechnology), mouse anti-human Runx2 (Santa Cruz Biotechnology), rabbit anti-human osteocalcin (Santa Cruz Biotechnology), rabbit anti-human GAPDH (Santa Cruz Biotechnology) or rabbit anti-human ß-tubulin (Santa Cruz Biotechnology) antibodies. The next day, the membranes were washed three times, and incubated with HRP-conjugated goat anti-rabbit or anti-mouse IgG antibody (Jackson Immunoresearch). For the Western-blots presented in Fig. 4A,B, signals were visualized with the chemiluminescence method (ECL plus western blotting detection reagent+, Santa Cruz Biotechnology, Inc.) and developed on X-ray films. For the Western-blots presented in Fi. 4 C, 6B and 8B signals were visualized by the chemiluminescence method (Clarity ECL, Biorad) and analyzed on the ChemiDoc Touch Imaging system with Image Lab Touch software (Biorad). Complete gels and different exposures of cropped presentations are provided in Figs S9–S11.

### Histochemical analysis

After 28 days of chondrogenic differentiation in 3D scaffold model, MSCs were harvested, washed twice in PBS, fixed by incubation in 10% formalin neutral buffer for 8 h at room temperature and embedded in paraffin. The paraffin blocks were cut into 4 µm-thick sections. The paraffin was removed by incubation in toluene and the sections were rehydrated by passage through a series of alcohol solutions of decreasing concentration. For alcian blue staining, deparaffined sections were rinsed in 3% acetic acid, stained with an alcian blue/3% acetic acid solution (pH = 2.5) (Sigma-Aldrich) and counterstained with Nuclear Fast Red solution (Sigma-Aldrich). For the analysis of ECM calcification, deparaffinized sections were stained with alizarin Red S solution (pH = 4.1) (Sigma-Aldrich). Sulfated proteoglycans were detected by counterstaining deparaffinized sections with 0.01% Fast Green solution (Sigma-Aldrich) and then incubating in 0.1 safranin-O solution (Sigma-Aldrich) before washing in absolute ethanol. Stained deparaffinized sections were dehydrated by passage through a series of alcohol solutions of graded concentrations, cleared in toluene and mounted.

### Statistical analysis

All experiments were replicated with cells samples from different specimens. Values are reported as means ± SD or box plots. Statistical analyses were performed with the Mann- Whitney U-test to assess the significance of differences between the two groups. All statistical analyses were performed with Prism v6 (Graphpad). P-values ≤ 0.05 was considered to be significant.

## Electronic supplementary material

Supplementary information
