# Publisher Correction: Differences in the intrinsic chondrogenic potential of equine umbilical cord matrix and cord blood mesenchymal stromal/stem cells for cartilage regeneration

**DOI:** 10.1038/s41598-020-69170-0

**Published:** 2020-07-21

**Authors:** Rodolphe Rakic, Bastien Bourdon, Magali Demoor, Stéphane Maddens, Nathalie Saulnier, Philippe Galéra

**Affiliations:** 1 Normandie Univ, Unicaen, Biotargen, Caen, 14000 Caen, France; 2 Vetbiobank, 69280 Marcy l’Etoile, France

Correction to: *Scientific Reports* 10.1038/s41598-018-28164-9, published online 14 September 2018

The original version of this Article contained errors in the figures. Figure 1 was truncated so that the right edge was missing. Additionally, in Figure 2, the graphs for UCM-MSCs and UCB-MSCs were presented as UCB-MSCs and UCM-MSCs respectively.

These errors have now been fixed in the HTML and PDF versions of the Article.

