# Supplementary material for: Differences in the intrinsic chondrogenic potential of equine umbilical cord matrix and cord blood mesenchymal stromal/stem cells for cartilage regeneration
Source: Sci Rep. 2018 Sep 14;8:13799. doi: 10.1038/s41598-018-28164-9 (PMC6138671; doi:10.1038/s41598-018-28164-9)
Supplement: Supplementary file 1 — Supplementary information [file 41598_2018_28164_MOESM1_ESM.pdf]

Differences in the intrinsic chondrogenic potential of equine umbilical cord matrix and cord blood mesenchymal stromal/stem cells for cartilage regeneration.

Rakic Rodolphe[1,2], Bourdon Bastien[1], Magali Demoor[1], Maddens Stéphane[2], Saulnier Nathalie[2†] and Galéra Philippe[1†*].

[1] NORMANDIE UNIV, UNICAEN, BIOTARGEN, 14000 CAEN, FRANCE.

[2] VETBIOBANK, 69280 Marcy l'Etoile, France.

[†] Contributed equally

[*] Corresponding author: philippe.galera@unicaen.fr

Dose effect of BMP-2 and TGF-ß1

	BMP-2 (ng/ml)	TGF-ß1 (ng/ml)	
1	-	-	Normoxia
2	5	5	
3	10	10	
4	-	-	
5	-	-	
6	5	5	
7	5	5	
8	10	10	
9	10	10	
10	-	-	Hypoxia
11	5	5	
12	10	10	
13	-	-	
14	-	-	
15	5	5	
16	5	5	
17	10	10	
18	10	10	

Chondrogenic conditions for the screening

	Treatment	
1	ICM	Normoxia
2	TGF-ß1 (10 ng/ml) + BMP-2 (50 ng/ml)	
3	TGF-ß3 (10 ng/ml)	
4	TGF-ß3 (10 ng/ml) + BMP-2 (50 ng/ml)	
5	BMP-6 (500 ng/ml)	
6	BMP-6 (500 ng/ml) + TGF-ß1 (10 ng/ml) + BMP-2 (50 ng/ml)	
7	BMP-6 (500 ng/ml) + TGF-ß3 (10 ng/ml)	
8	Stempro +supplement (TermoFisher)	
9	Poietics complete (Lonza)	
10	Poietics complete + TGF-ß3 (10 ng/ml)	
11	ICM	Hypoxia
12	TGF-ß1 (10 ng/ml) + BMP-2 (50 ng/ml)	
13	TGF-ß3 (10 ng/ml)	
14	TGF-ß3 (10 ng/ml) + BMP-2 (50 ng/ml)	
15	BMP-6 (500 ng/ml)	
16	BMP-6 (500 ng/ml) + TGF-ß1 (10 ng/ml) + BMP-2 (50 ng/ml)	
17	BMP-6 (500 ng/ml) + TGF-ß3 (10 ng/ml)	
18	Stempro +supplement (TermoFisher)	
19	Poietics complete (Lonza)	
20	Poietics complete + TGF-ß3 (10 ng/ml)	

Supplemental table I: lists of the growth factors concentrations and chondrogenic conditions used for the screening.

Gene	Primer	sequence 5'-3'
Actb	forward	GATGATGATATCGCCGCGCTC
	reverse	TGCCCCACGTATGAGTCCTT
Col1a1	forward	TGCCGTGACCTCAAGATGTG
	reverse	CGTCTCCATGTTGCAGAAGA
Col2a1	forward	GGCAATAGCAGGTTCACGTACA
	reverse	CGATAACAGTCTTGCCCCACTT
Col10a1	forward	GCACCCCAGTAATGTACACCTATG
	reverse	GAGCCACACCTGGTCATTTTC
Acan	forward	ACACGGATGGTGTCCTCTTC
	reverse	CTCAGTCCACGGGTTACGAT
Runx-2	forward	GCAGTTCCCAAGCATTTCAT
	reverse	CACTCTGGCTTTGGGAAGAG
Mmp13	forward	TGAAGACCCGAACCCTAAACAT
	reverse	GAAGACTGGTGATGGCATCAAG
Sox9	forward	CAAGAAGGACCACCCGGACTA
	reverse	GGAGATGTGTGTCTGCTCCGT
Alpl	forward	GACATGACCTCCCAGGAAGA
	reverse	GCAGTGAAGGGCTTCTTGTC
Htra1	forward	GGACTTCATGTTTCCCTCAA
	reverse	GTTCTGCTGAACAAGCAACA
Mcam	forward	GCTCCGTGTGTACAAAGCTCC
	reverse	GGGACGACTGAATGTGGACT
Ppia	forward	CCCTACCGTGTTCTTCGACA
	reverse	GTGAAGTCACCACCCTGACA
Bglap	forward	TCCTTTGGGGTTTGGCCTAC
	reverse	GCCTGTGAGACAAAGGAGGG
Spp1	forward	GAGACACGTATGATGGCCGA
	reverse	GCTGTCCCAATCAGAAGCCA

Supplemental table II: Primers used in RT-qPCR analysis.

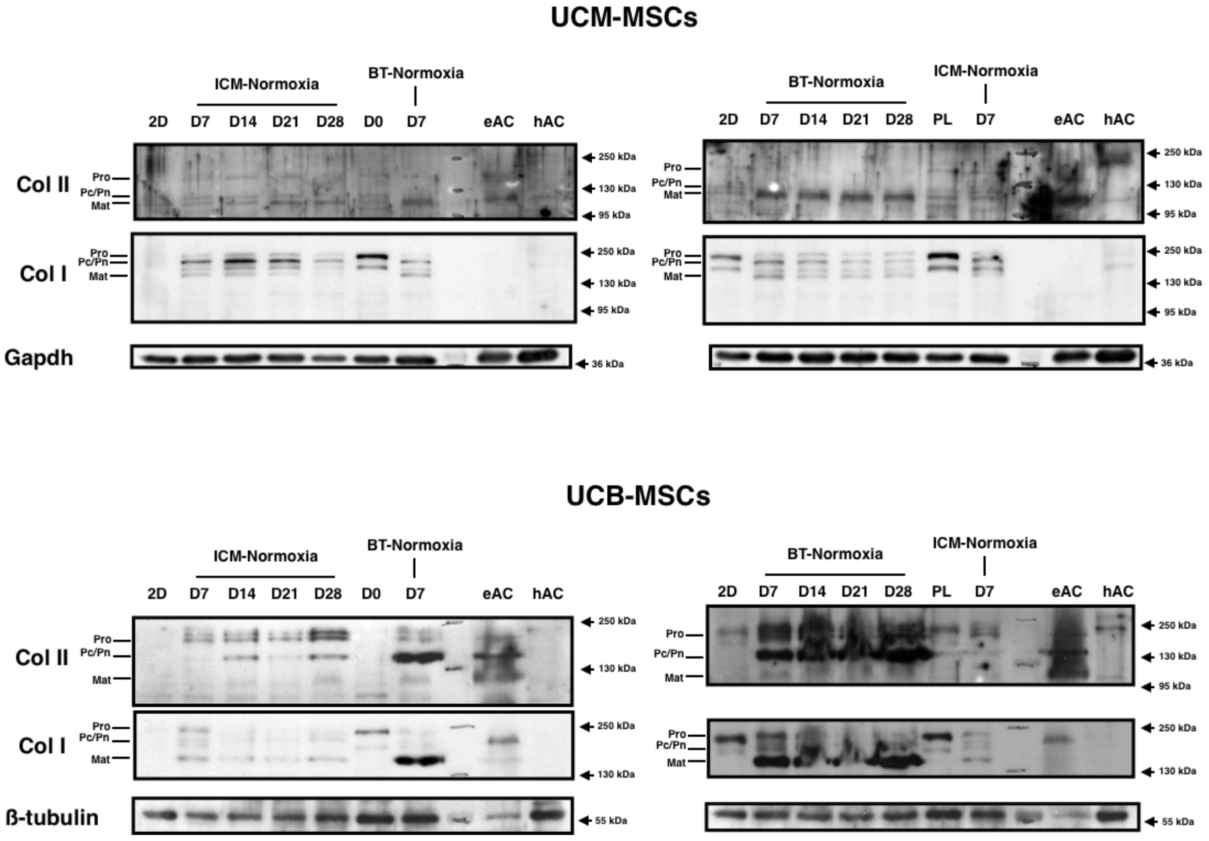

Supplemental figure 1: Comparison of ICM and BT treatments on UCM-MSCs and UCB-MSCs.

UCM-MSCs and UCB-MSCs were cultured in type I/III collagen scaffolds at 21% O_2 during 7, 14, 21 and 28 days in ICM supplemented with BMP-2 (50 ng/ml) and TGF-ß1 (10 ng/ml). Protein extracts were analyzed in western-blots for type II and type I collagen. Representative blots of UCM-MSCs (n= 5) and UCB-MSMs (n= 3) are shown. Blots were obtained from 2 independent experiments and 2 western-blots of 2 gels each. Different levels of type II and type I collagen maturation forms are indicated such as type II procollagen (pro), without C- or N- terminal propeptides (Pc/Pn) and the mature doubly cleaved form (mat).

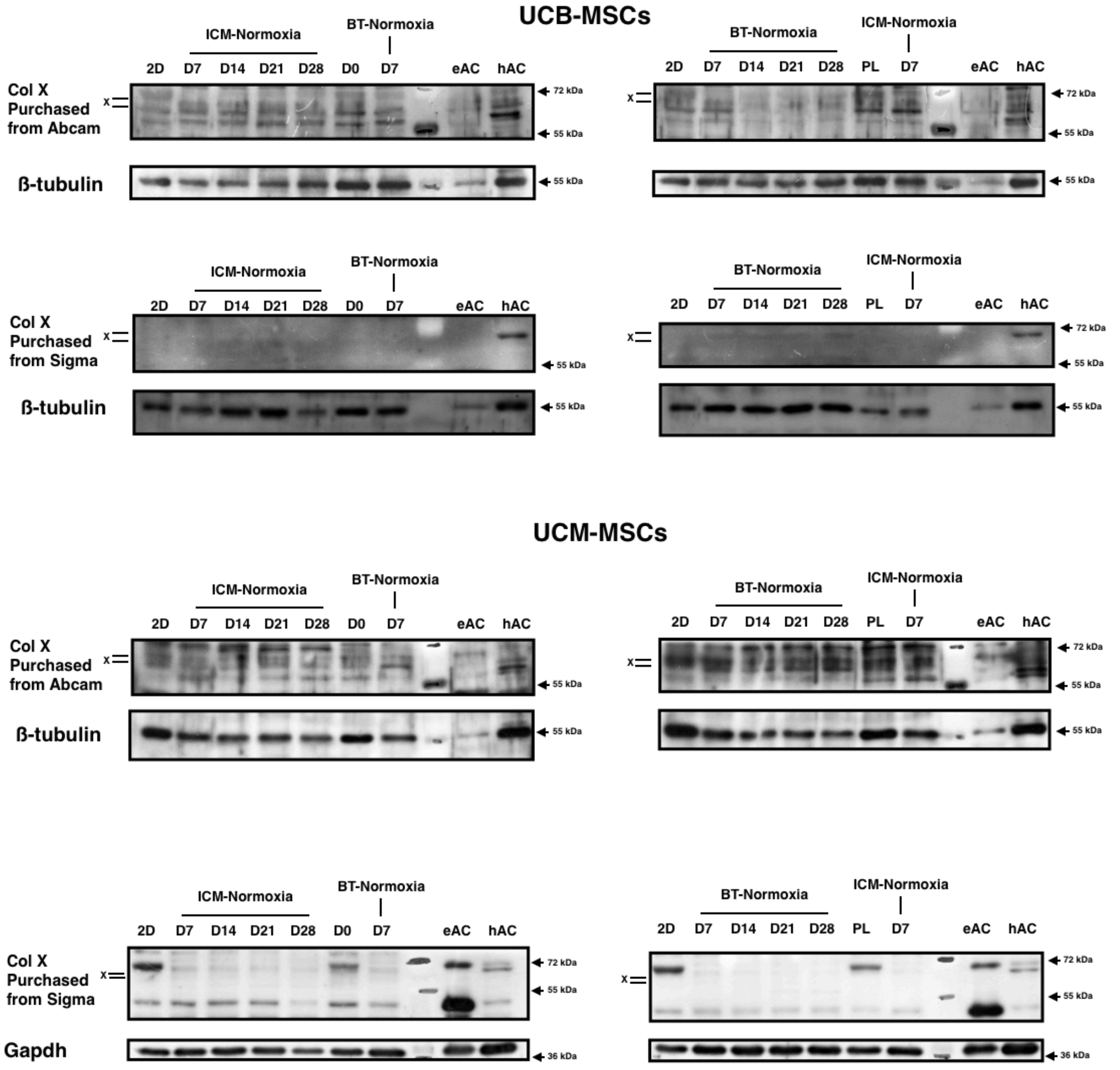

Supplemental figure 2: Analysis of type X collagen expression in UCB-MSCs and UCM-MSCs protein extracts.

UCB-MSCs and UCM-MSCs were cultured in type I/III collagen scaffolds at 21% O_2 during 7, 14, 21 and 28 days in ICM supplemented with BMP-2 (50 ng/ml) and TGF-ß1 (10 ng/ml). Protein extracts were analyzed in western-blots for type X collagen. Representative blots of UCM-MSCs (n= 5) and UCB-MSMs (n= 3) are shown. Blots were obtained from 4 independent experiments and 4 western-blots of 2 gels each. Two forms of type X collagen should be observed corresponding to the long form (66 kDa) and signal peptide clived form (64 kDa).

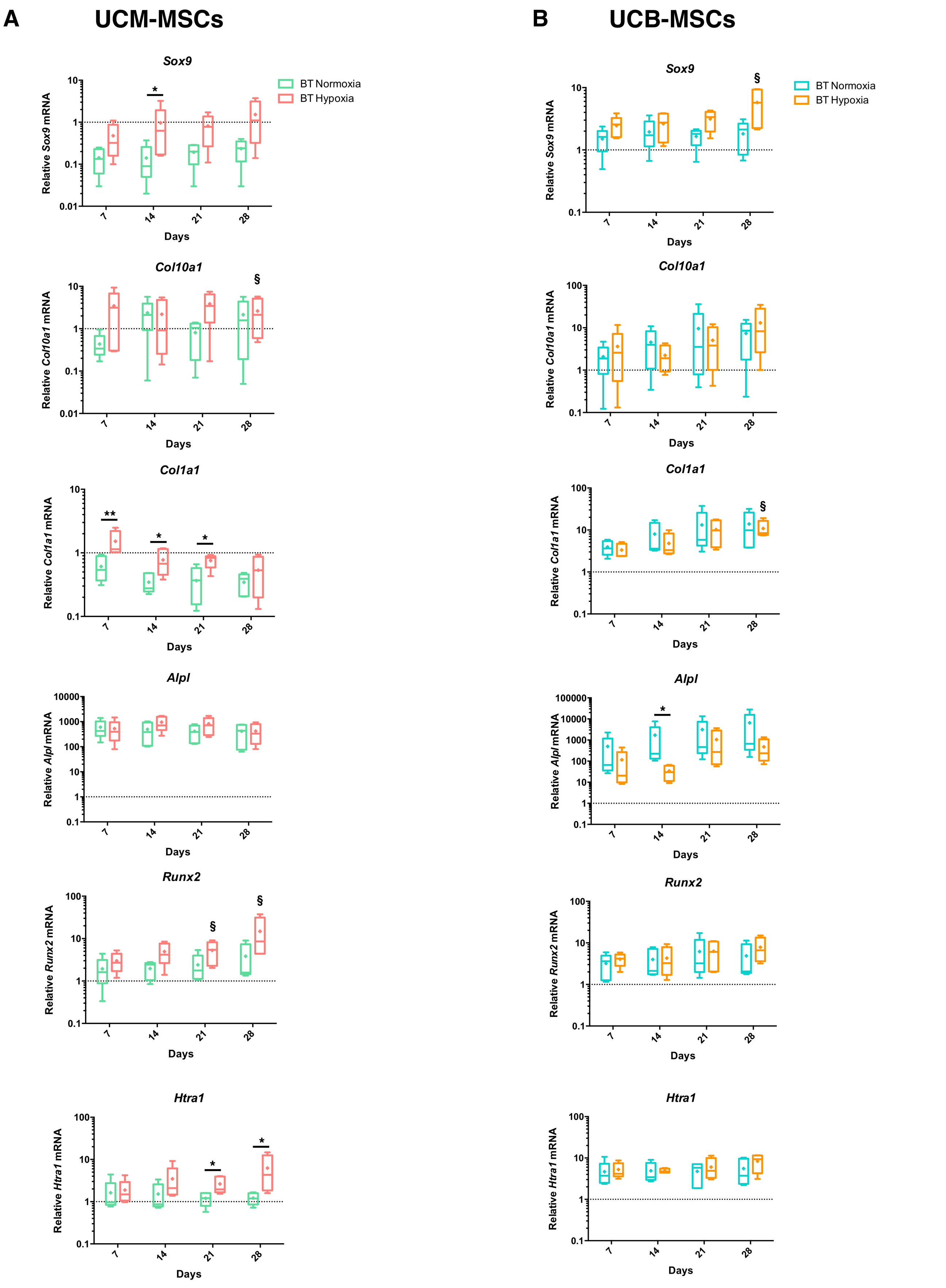

Supplemental figure 3: Differential gene expression modulation under hypoxia during a kinetic

culture under BMP-2 and TGF-ß1 treatment of UCM-MSCs and UCB-MSCs.

UCM-MSCs and UCB-MSCs were cultured in type I/III collagen scaffolds at 21% or 3-5% O_2 during 7, 14, 21 and 28 days in ICM supplemented with BMP-2 (50 ng/ml) and TGF-ß1 (10 ng/ml). Relative mRNA expression was determined by RT-qPCR. All results for each MSCs source were normalized versus untreated MSCs incubated during 1 h in type I/III collagen scaffolds at 37°C and presented as the relative expression of each gene. Box-plots represent five independent experiments for each MSCs source. Statistically significant differences between normoxia and hypoxia conditions were determined using a Mann Whitney test. *: Hypoxia versus normoxia at the same time (*$p < 0.05$, **$p < 0.01$, ***$p < 0.001$). §: Hypoxia versus normoxia at 7 days (§$p < 0.05$, §§$p < 0.01$, §§§$p < 0.001$).

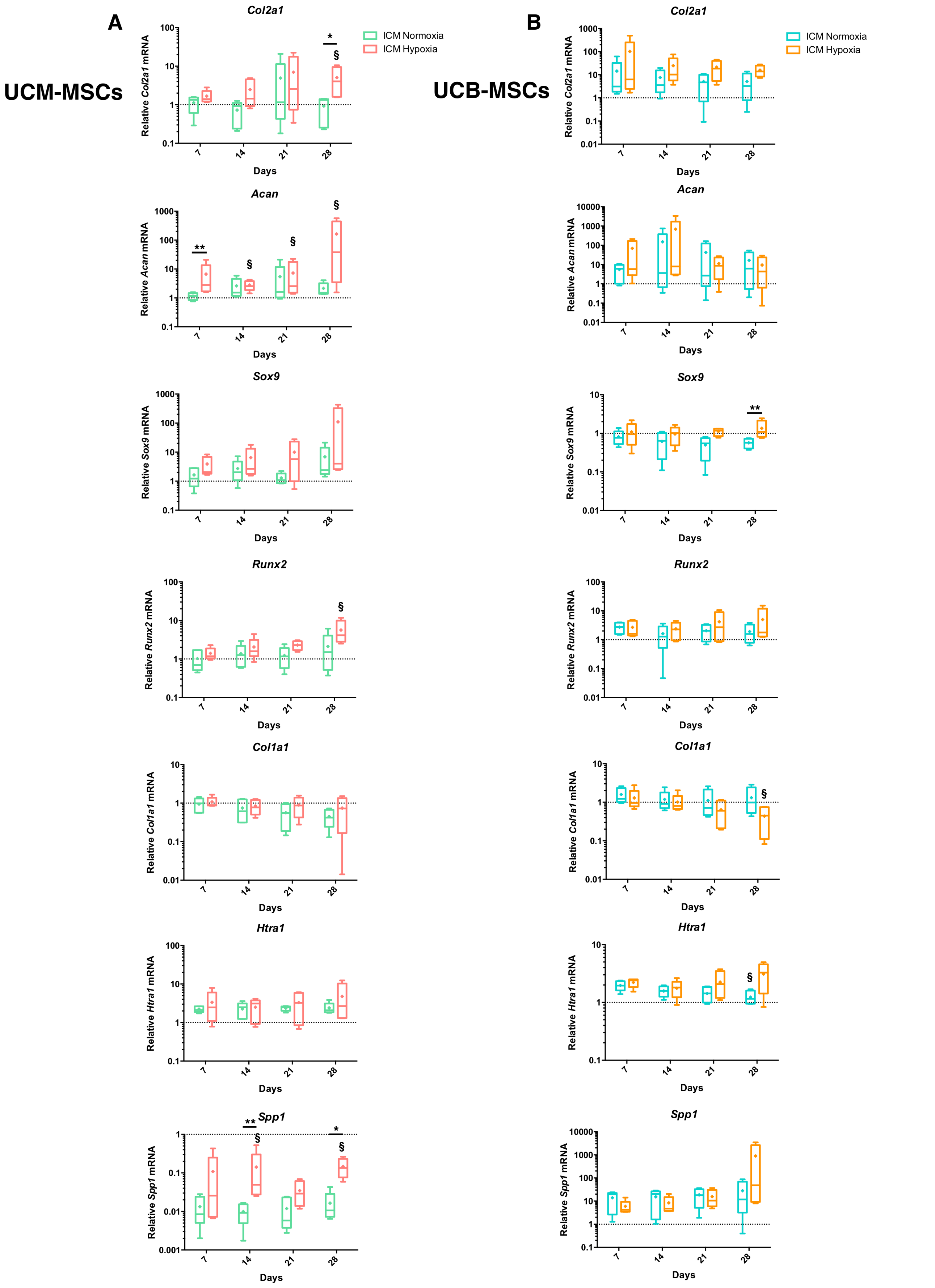

Supplemental figure 4: Differential gene expression modulation by hypoxia in a kinetic culture of

UCB-MSCs and UCM-MSCs cultured without growth factors.

UCM-MSCs and UCB-MSCs were cultured in type I/III collagen scaffolds at 21% or 3-5% O_2 during 7, 14, 21 and 28 days in ICM in absence of growth factors. Relative mRNA expression was determined by RT-qPCR. All results for each MSCs source were normalized versus untreated MSCs incubated during 1 h in collagen type I/III scaffolds at 37°C and presented as the relative expression of each gene. Box-plots represent five independent experiments for each MSCs source. Statistically significant differences between normoxia and hypoxia conditions were determined using a Mann Whitney test. *: Hypoxia versus normoxia at the same time (*$p < 0.05$, **$p < 0.01$, ***$p < 0.001$). §: Hypoxia versus normoxia at 7 days (§$p < 0.05$, §§$p < 0.01$, §§§$p < 0.001$).

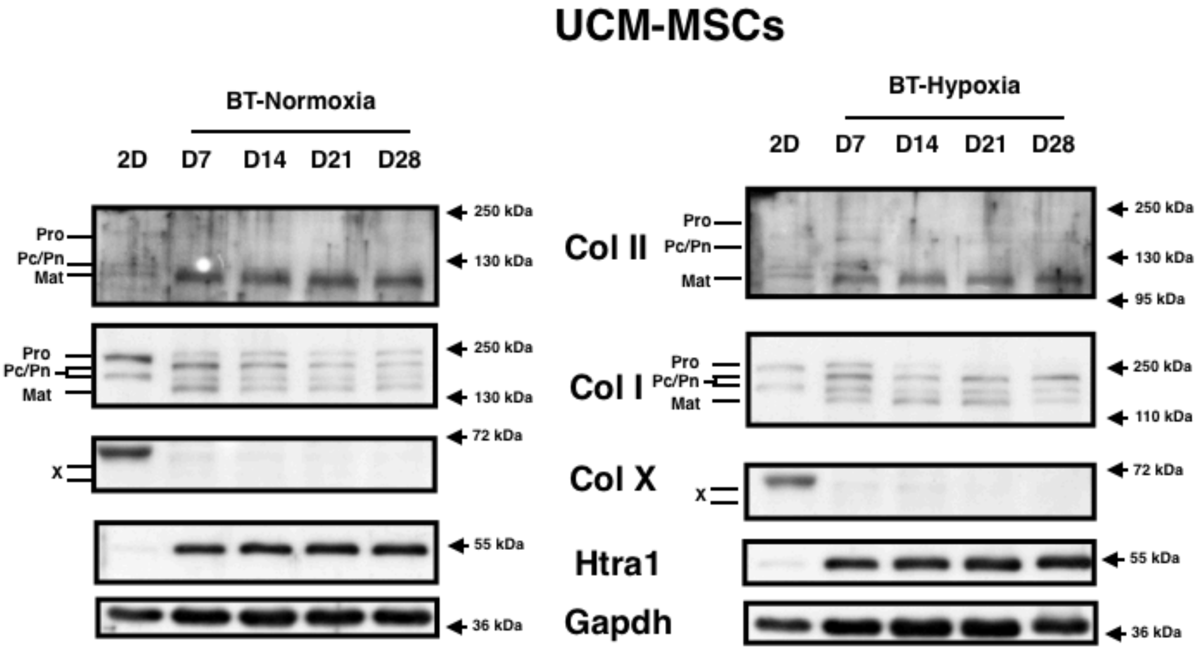

Supplemental figure 5: Effect of hypoxia on matrix synthesis by UCM-MSCs under growth factors treatment.

UCM-MSCs were cultured in type I/III collagen scaffolds at 21% or 3-5% O_2 during 7, 14, 21 and 28 days in ICM supplemented with BMP-2 (50 ng/ml) and TGF-ß1 (10 ng/ml). Protein extracts were analyzed in western-blots for types II, I and X collagens and Htra1. Representative blots of UCM-MSCs (n= 5) are shown. The images were obtained from one experiment and one western-blot of 2 gels. Different levels of type II and type I collagen maturation forms are indicated such as type II procollagen (pro), with only C- or N- terminal propeptides (Pc/Pn) and the mature doubly cleaved form (mat).

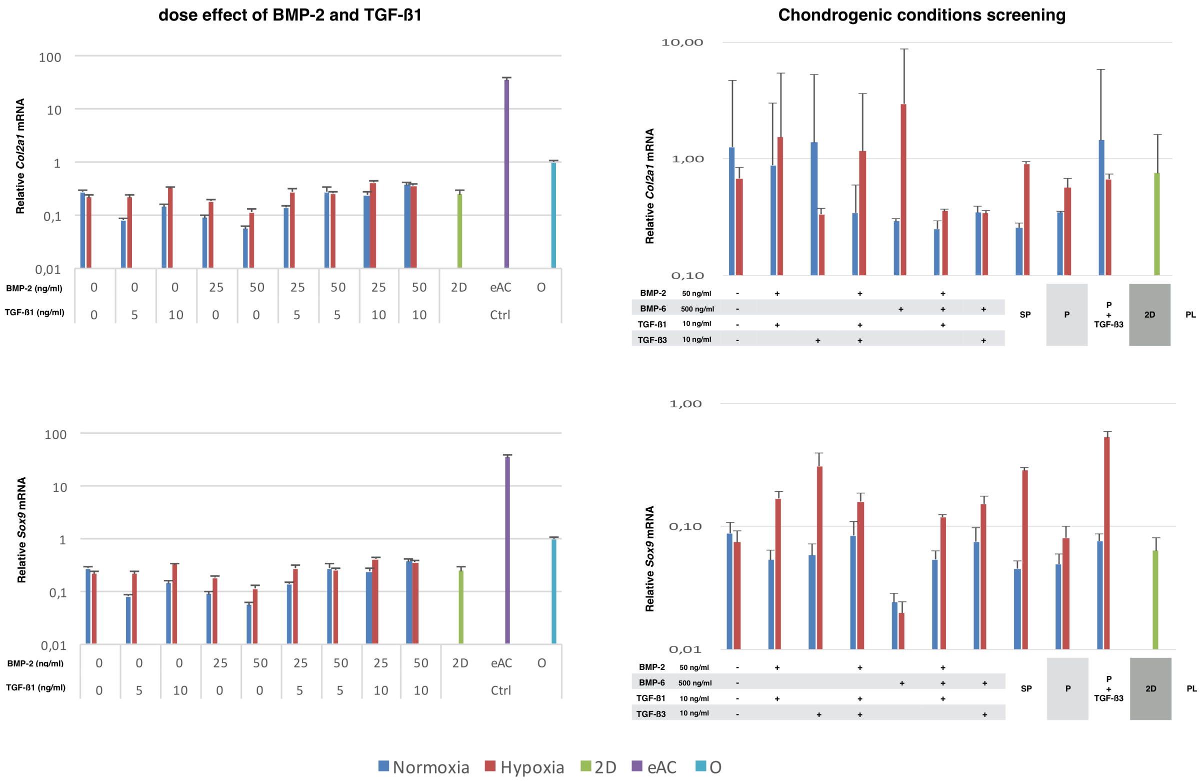

Supplemental figure 6: Effect of different growth factors and chondrogenic culture conditions screening on chondrogenesis induction in UCM-MSCs.

UCM-MSCs were cultured in type I/III collagen scaffolds at 21% or 3-5% O_2 during 14 days in various chondrogenic media and growth factors presented in Supp. table I. Relative mRNA amounts encoding *Col2a1* and *Sox9* were analyzed by RT-qPCR and shown. mRNA extracts obtained from equine articular chondrocytes (eAC) released from cartilage after overnight enzymatic digestion and equine primary (P0) osteoblasts (O) are used as controls. Monolayer cultured MSCs were used as a control for the undifferentiated status. All results were normalized versus untreated MSCs incubated during 1 h in type I/III collagen scaffolds at 37°C and presented as the relative expression of each gene. A representative experiment of two independent experiments is presented.

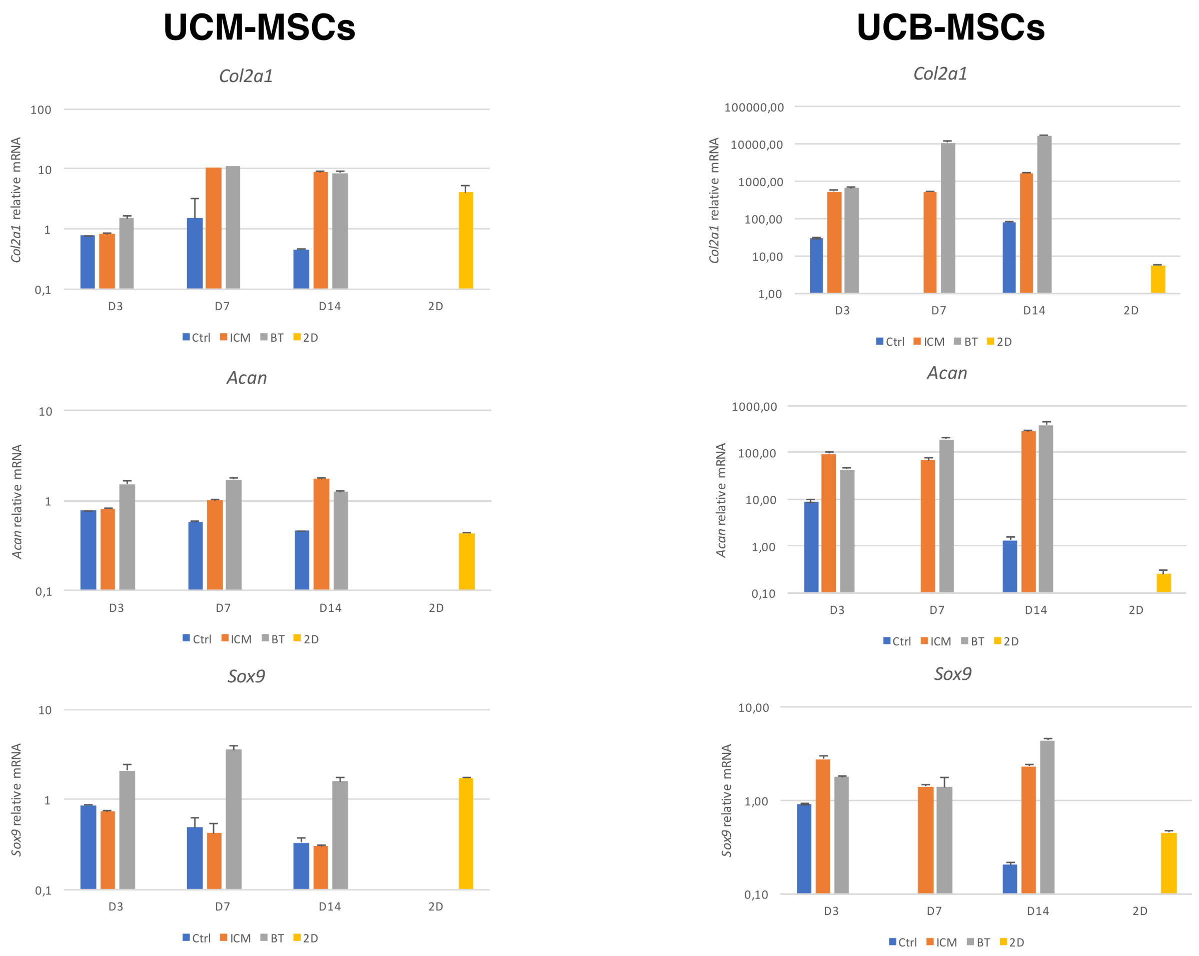

Supplemental figure 7: UCM-MSCs and UCB-MSCs chondrogenic differentiation in a hanging-drop cell culture model.

UCM-MSCs and UCB-MSCs were cultured in a hanging-drop model during 14 days in amplification medium (Ctrl) or in ICM with or without BT treatment. Relative mRNA amounts encoding *Col2a1*, *Acan* and *Sox9* were determined by RT-qPCR and shown. All results for each MSCs source were normalized versus a 24 h hanging drop culture before treatment (D0) and presented as the relative expression of each gene. A representative experiment of two independent experiments is presented.

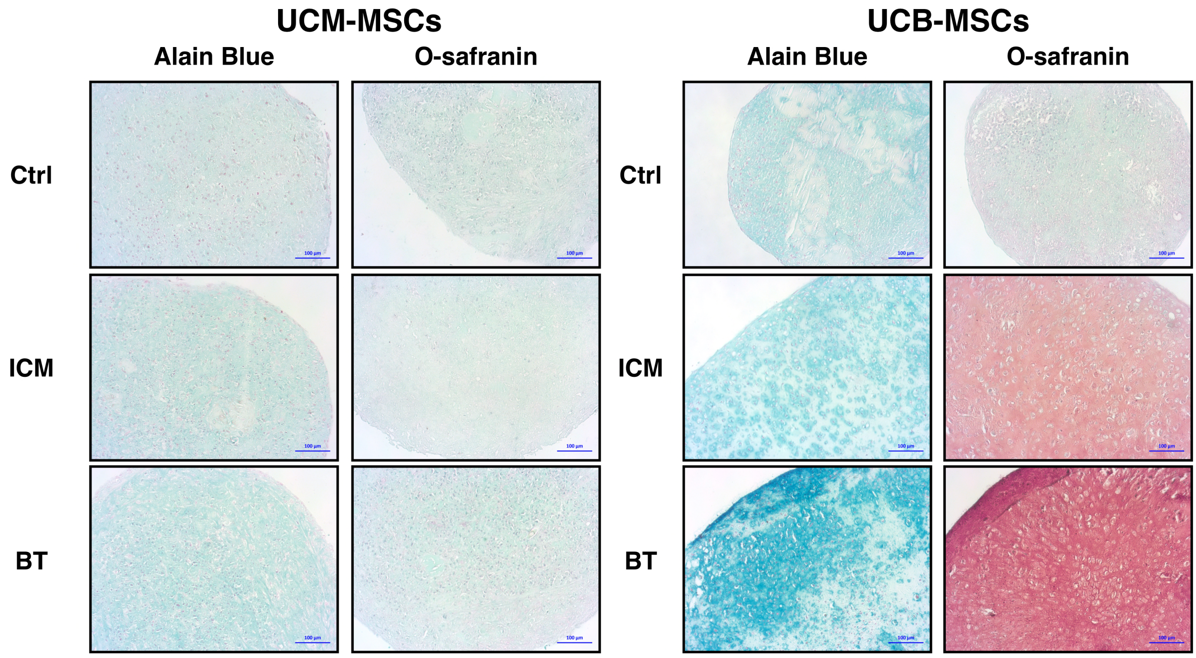

Supplemental figure 8: Histochemistry of UCM-MSCs and UCB-MSCs chondrogenic differentiation in a micro-pellet cell culture model.

UCM-MSCs and UCB-MSCs were cultured as micro-pellets during 28 days in amplification medium (Ctrl) or in ICM with or without BT treatment. The quality of the ECM synthesized was evaluated by Alcian blue and O-safranin stainings. A representative example of two independent experiments is shown (magnification: ×20, scale bar: 100 µm).

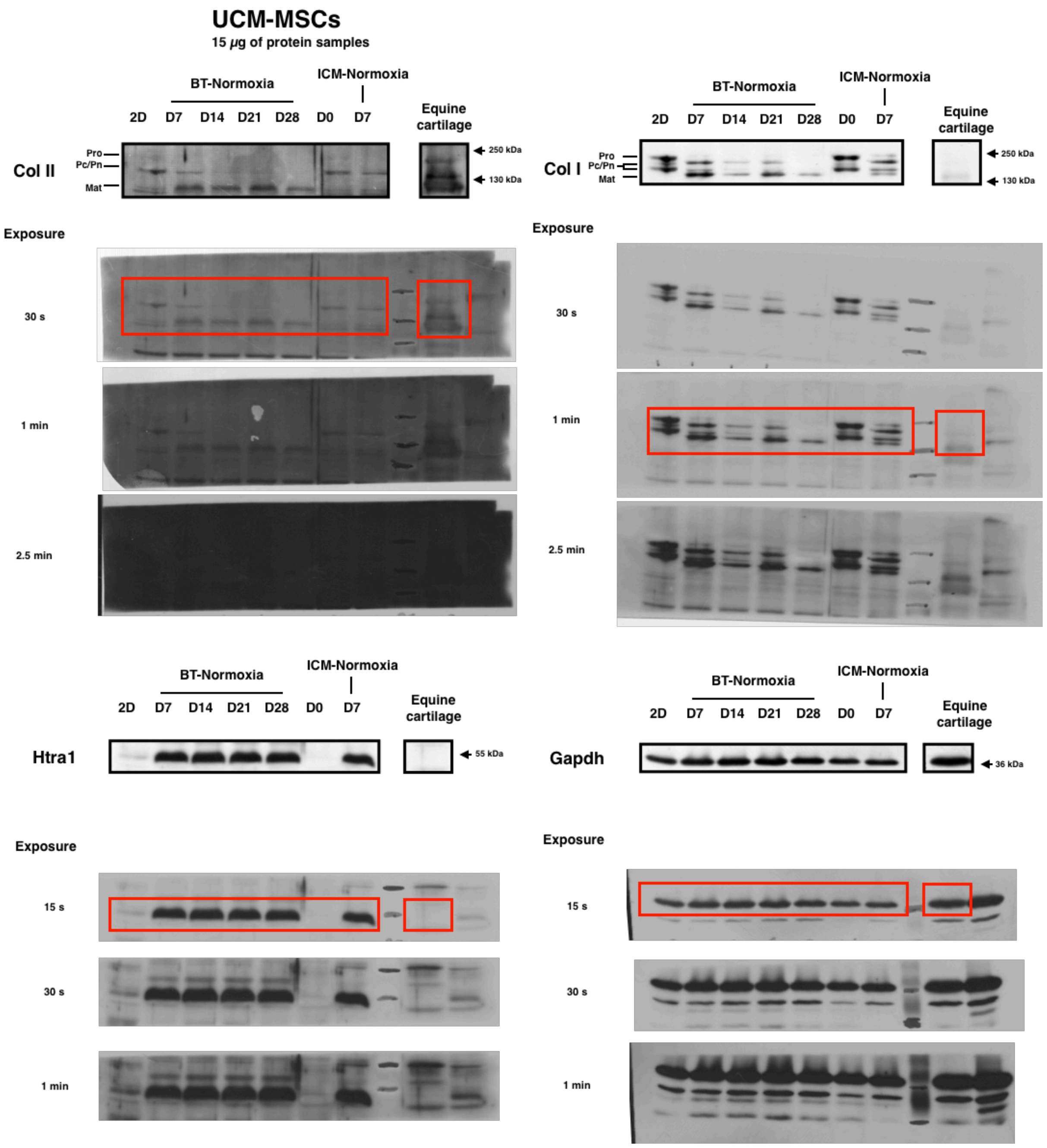

Supplemental figure 9: Complete gel and different exposure times of the autoradiographic films for the western-blots.

For the western-blots presented in figure 4A, different exposures of one PVDF membrane were done as shown. The cropped images are highlighted in the red lines.

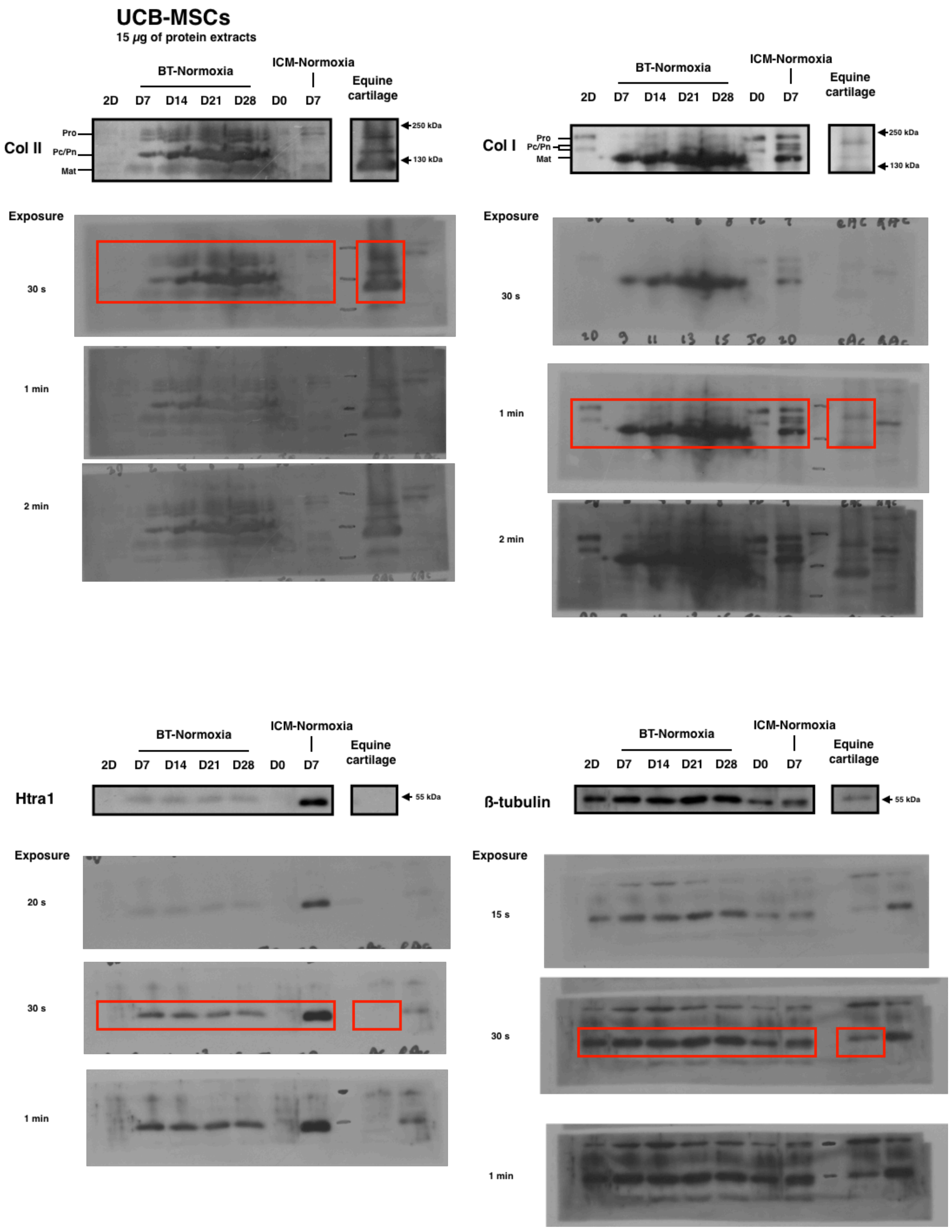

Supplemental figure 10: Complete gel and different exposure times of the autoradiographic films for the western-blots.

For the western-blots presented in figure 4B, different exposures of one PVDF membrane were performed as presented. The cropped images are highlighted in the red lines.

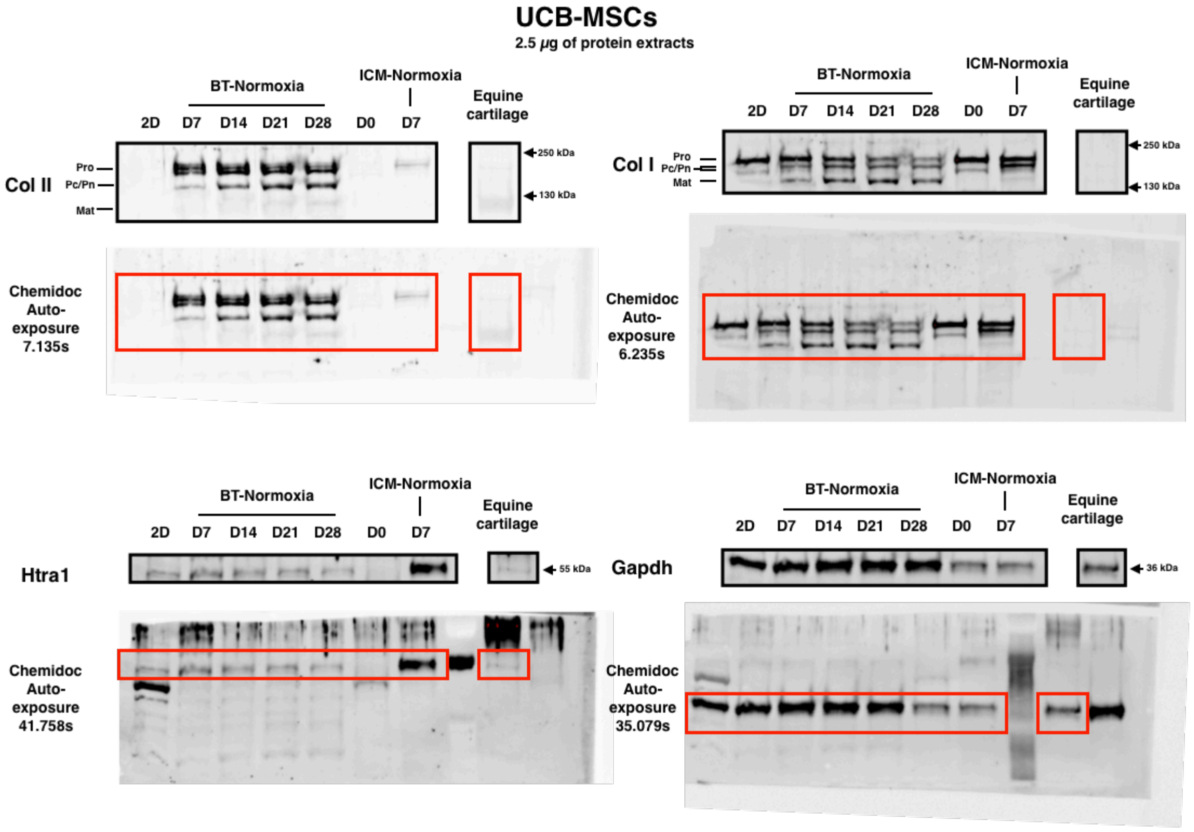

Supplemental figure 11: Complete gel and PVDF membranes analyzed in the western-blots.

For the western-blots presented in figure 4C, one PVDF membrane was analyzed by the ChemiDoc MP Imaging System (Bio-Rad) with automatic exposure parameters. The cropped images are highlighted in the red lines.

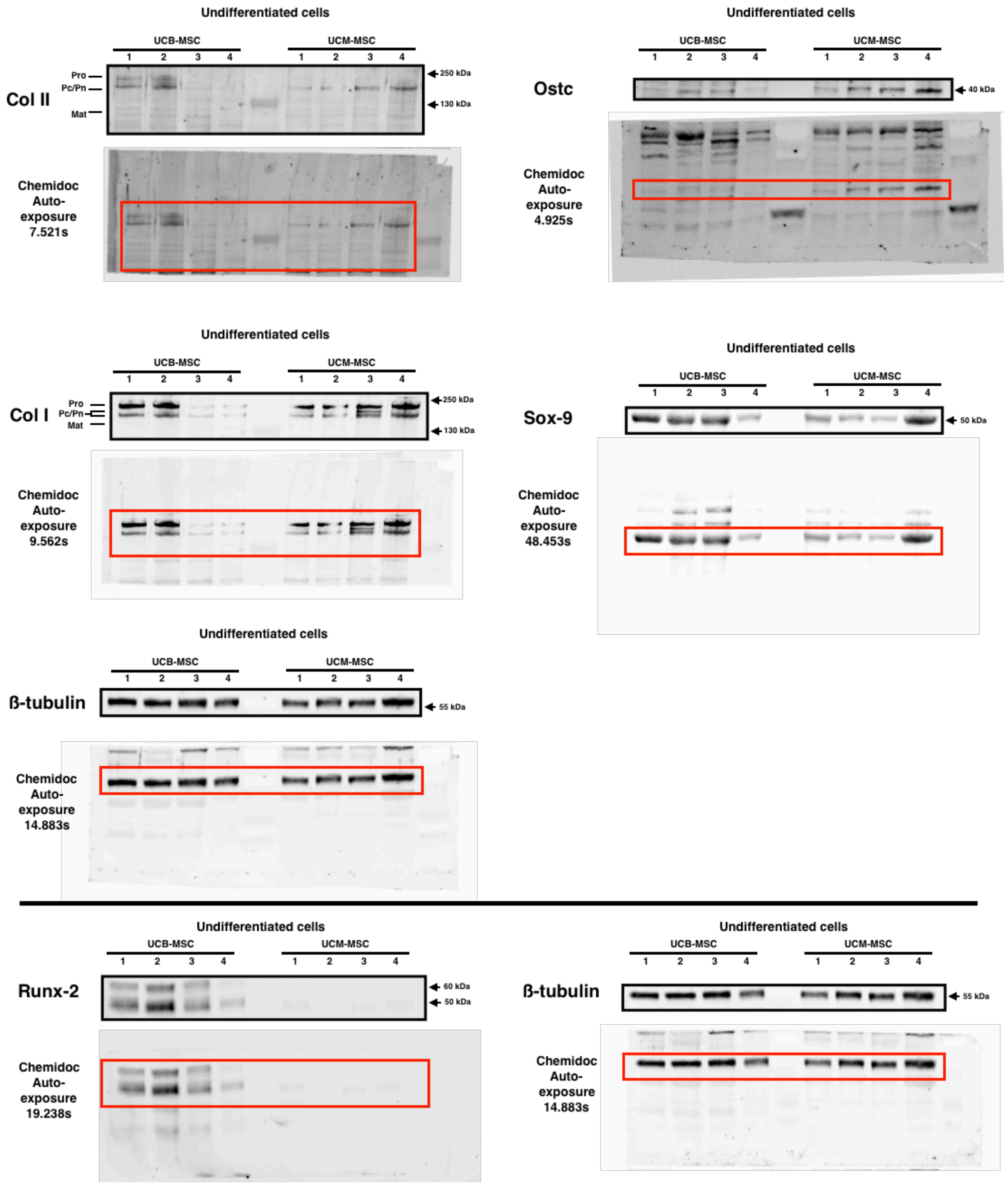

Supplemental figure 12: Complete gel and the polyvinylidene difluoride membranes analyzed in the western-blots.

For the western-blots presented in figure 6B, two PVDF membranes were analyzed by the ChemiDoc MP Imaging System (Bio-Rad) with automatic exposure parameters. The horizontal black line separates different western-blot membranes from two series, this is the reason why the ß-tubulin was presented twice, one presentation for each series. The cropped images are highlighted in the red lines.

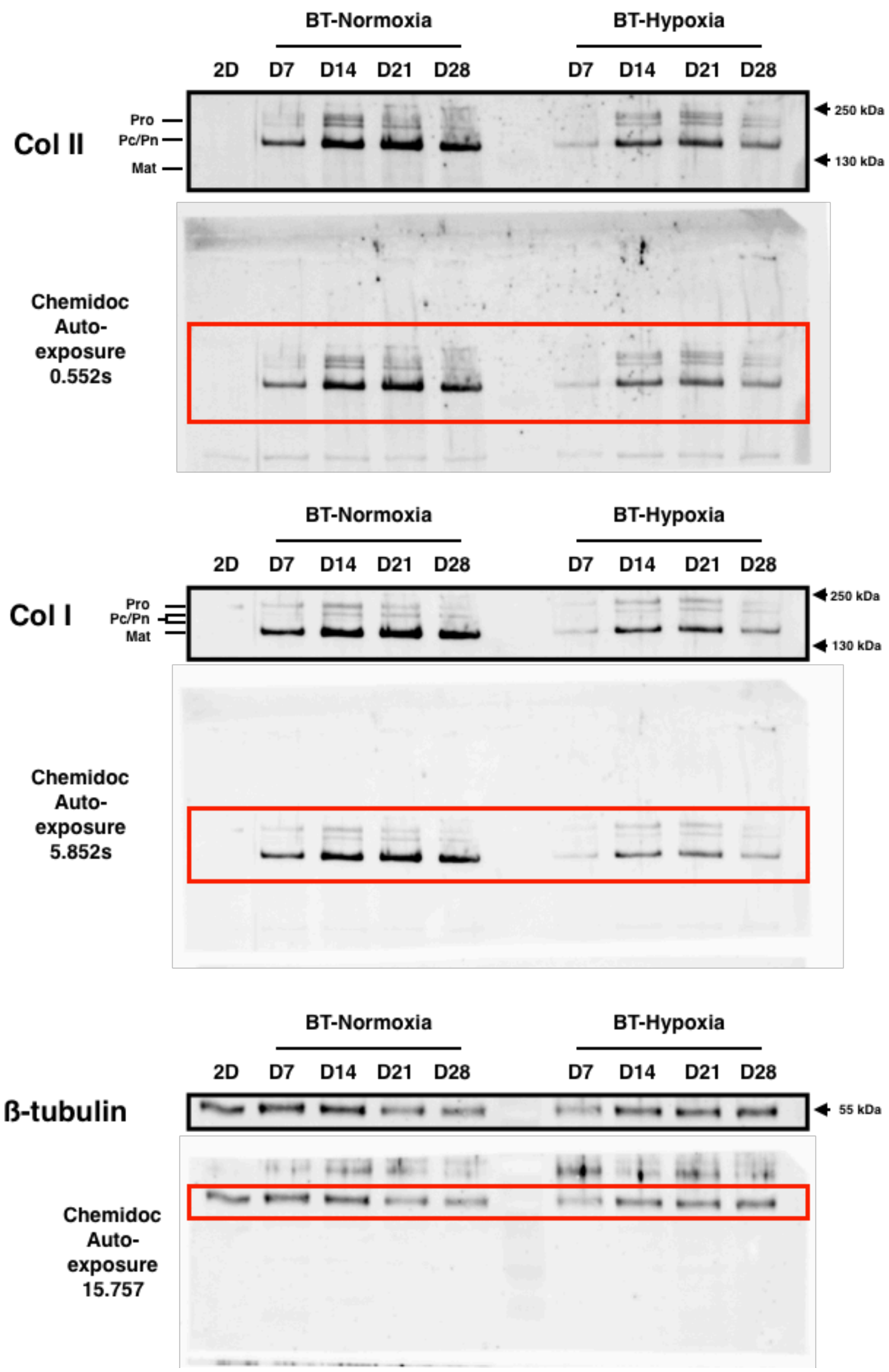

Supplemental figure 13: Complete gel and the polyvinylidene difluoride membranes analyzed in the western-blots.

For the western-blots presented in supplemental figure 8B, one PVDF membrane was analyzed by the ChemiDoc MP Imaging System (Bio-Rad) with automatic exposure parameters. The cropped images are highlighted in the red lines.

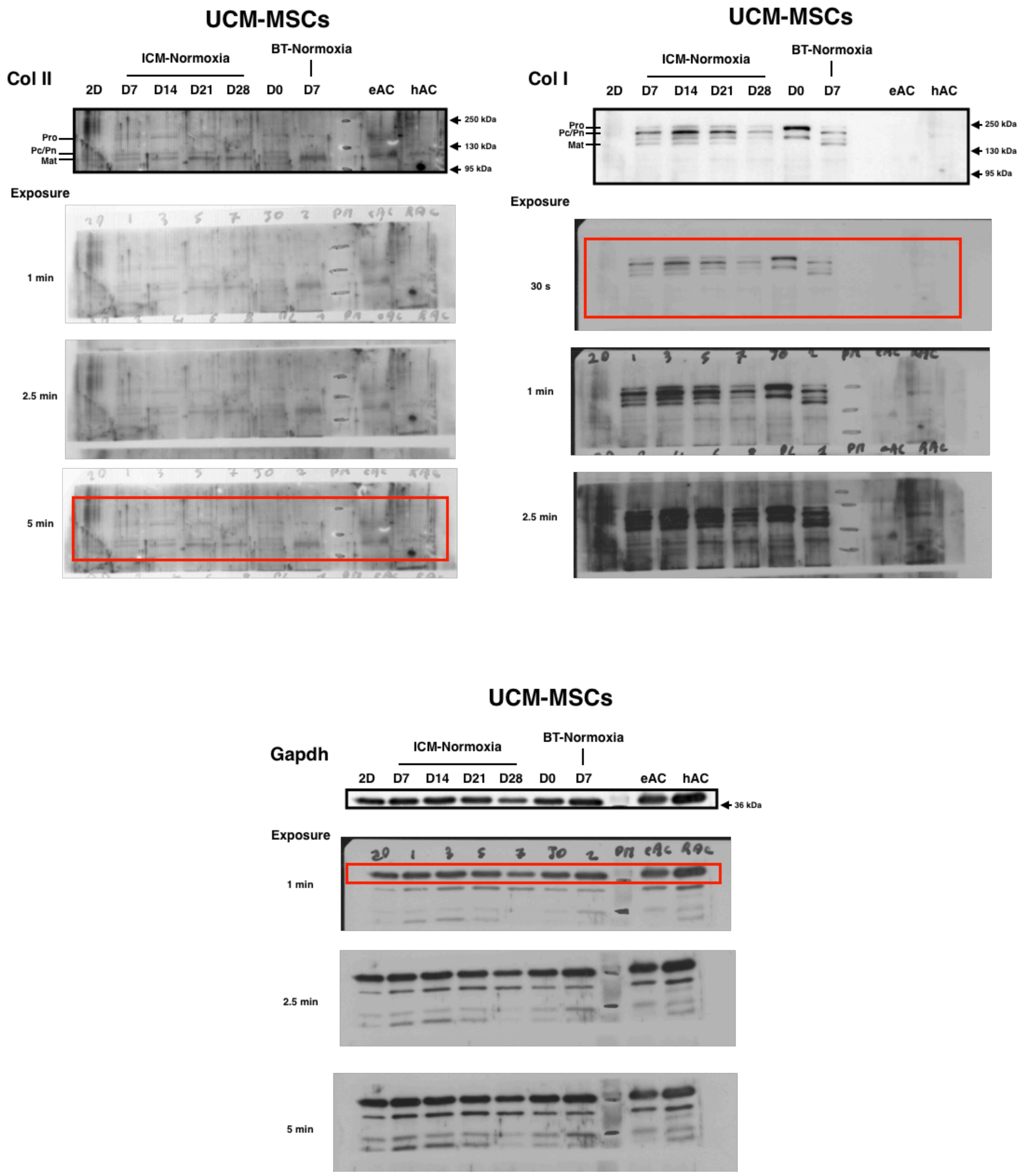

Supplemental figure 14: Complete gel and the polyvinylidene difluoride membranes analyzed in the the western-blots.

For the western-blots presented in supplemental figure 1 (top left), the blots were obtained from 1 independent experiment and 1 western-blot of 1 gel. The cropped images are highlighted in the red lines.

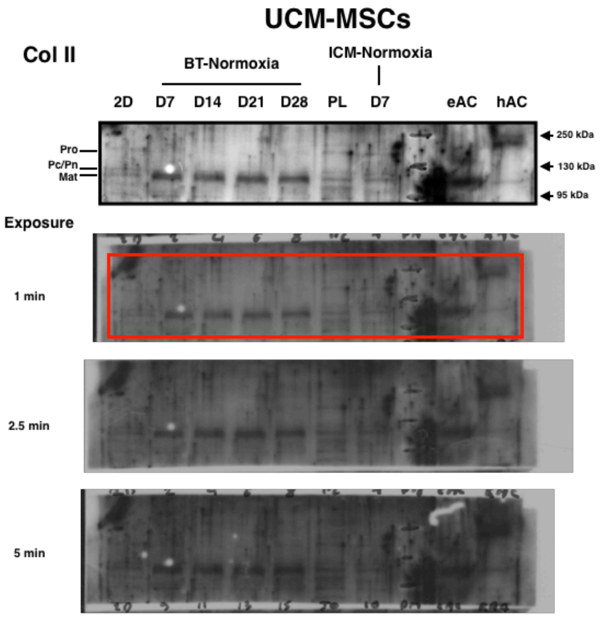

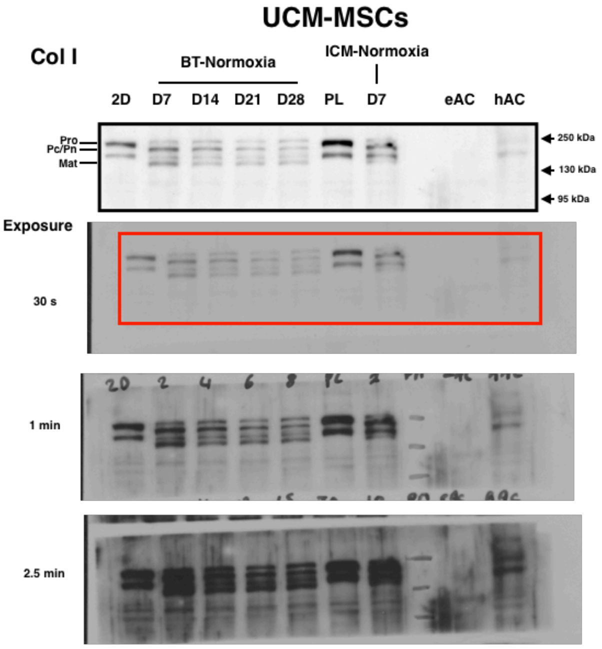

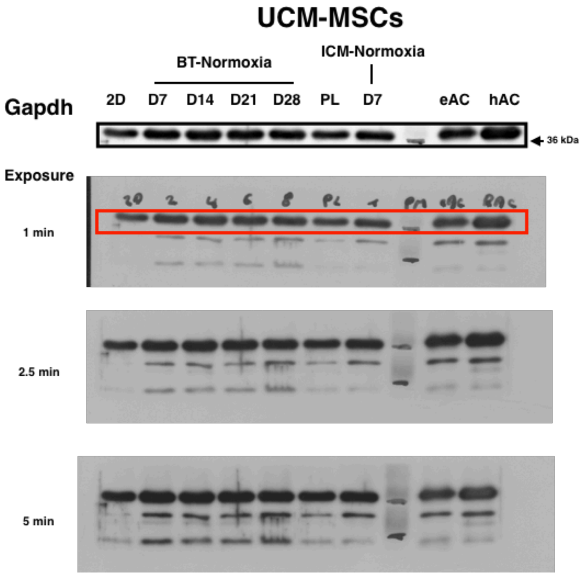

Supplemental figure 15: Complete gel and the polyvinylidene difluoride membranes analyzed in the western-blots.

For the western-blots presented in supplemental figure 1 (top right), the blots were obtained from 1 independent experiment and 1 western-blot of 1 gel. The cropped images are highlighted in the red lines.

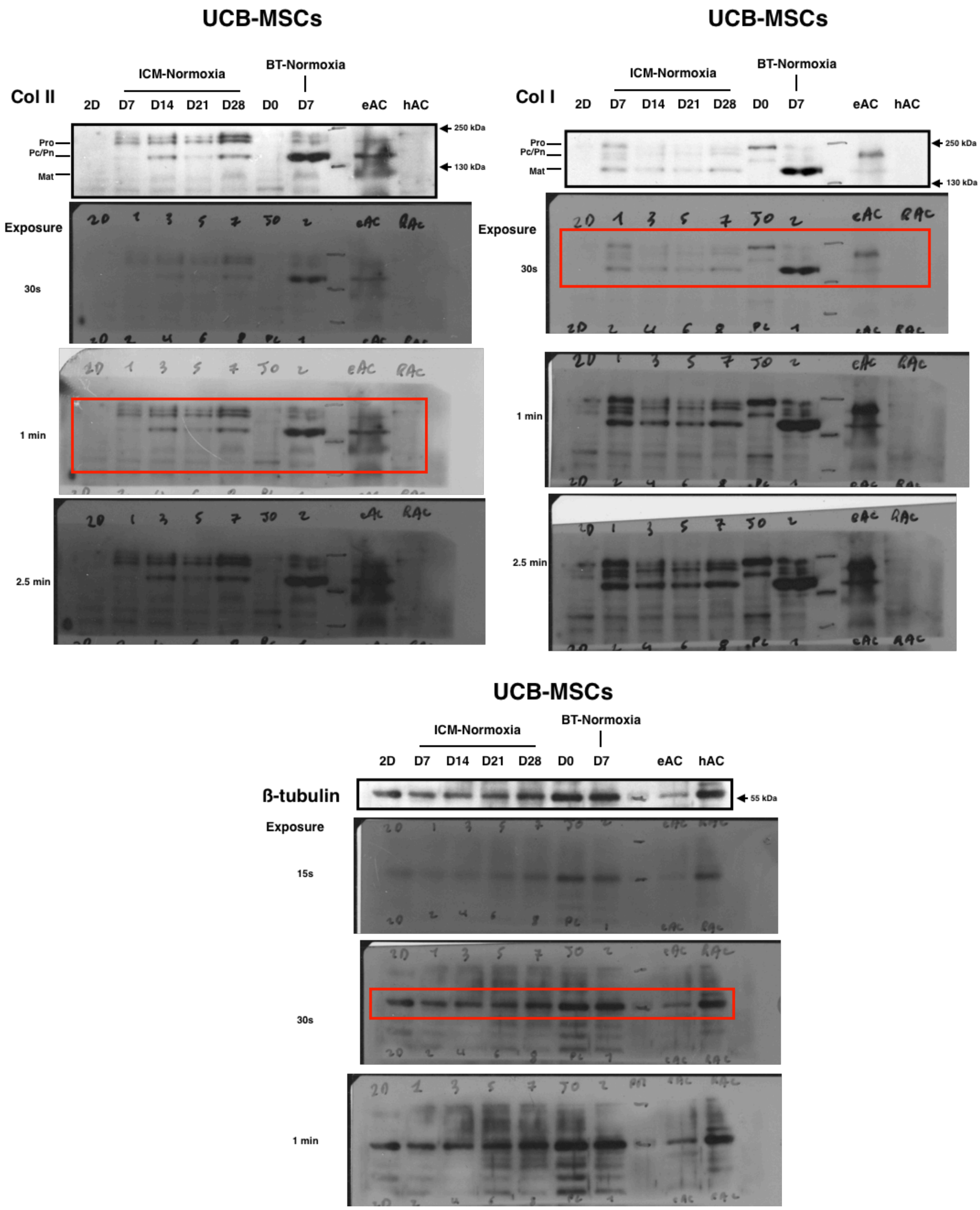

Supplemental figure 16: Complete gel and the polyvinylidene difluoride membranes analyzed in the western-blots.

For the western-blot presented in supplemental figure 1 (bottom left), the blots were obtained from 1 independent experiment and 1 western-blot of 1 gel. The cropped images are highlighted in the red lines.

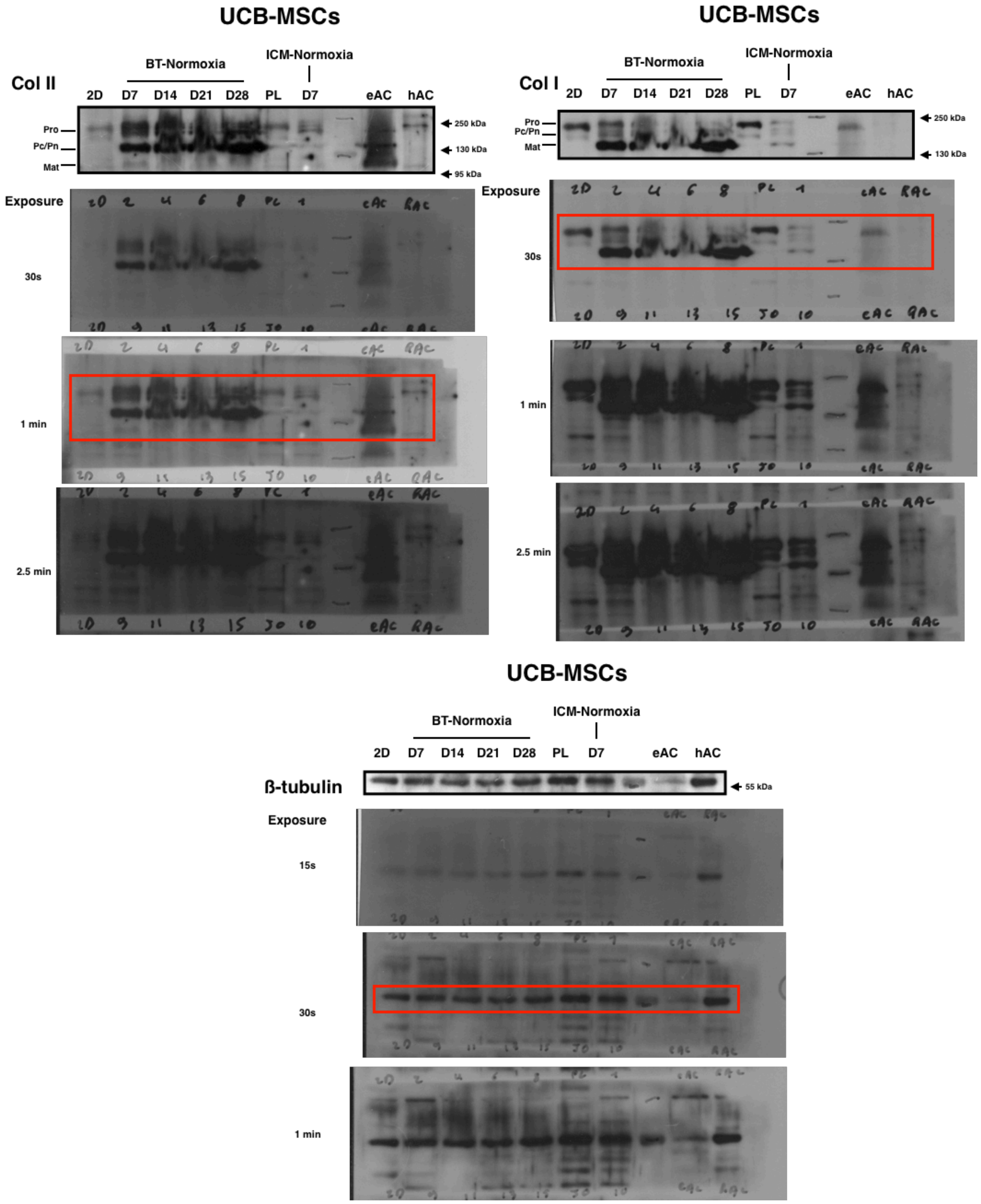

Supplemental figure 17: Complete gel and the polyvinylidene difluoride membranes analyzed in the western-blots.

For the western-blots presented in supplemental figure 1 (bottom right), the blots were obtained from 1 independent experiment and 1 western-blot of 1 gel. The cropped images are highlighted in the red lines.

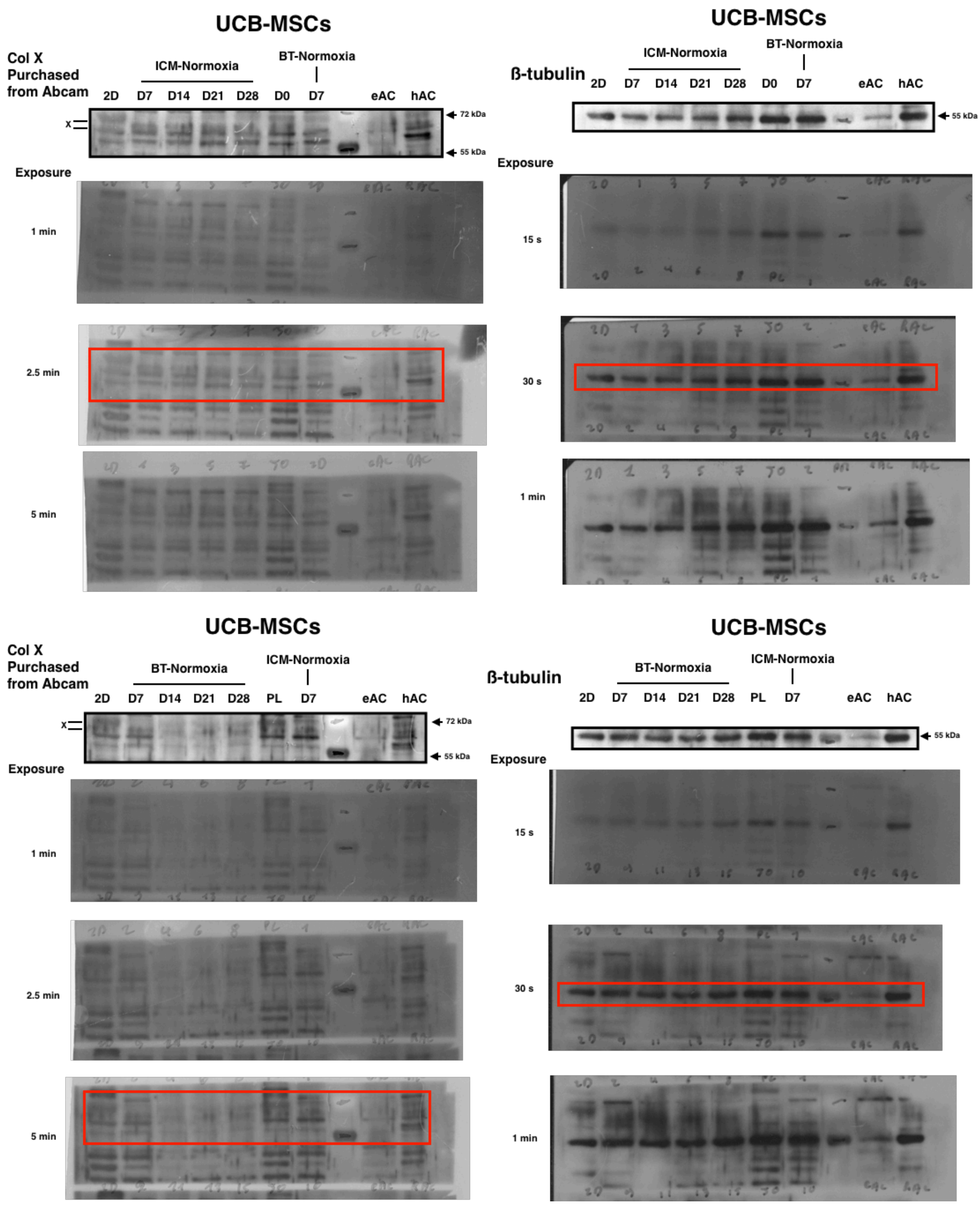

Supplemental figure 18: Complete gel and the polyvinylidene difluoride membranes analyzed in the western-blots.

For the western-blots (WB) presented in supplemental figure 2 (top WB for UCB-MSCs), the blots were obtained from 1 independent experiment and 1 western-blot of 2 gels. The cropped images are highlighted in the red lines.

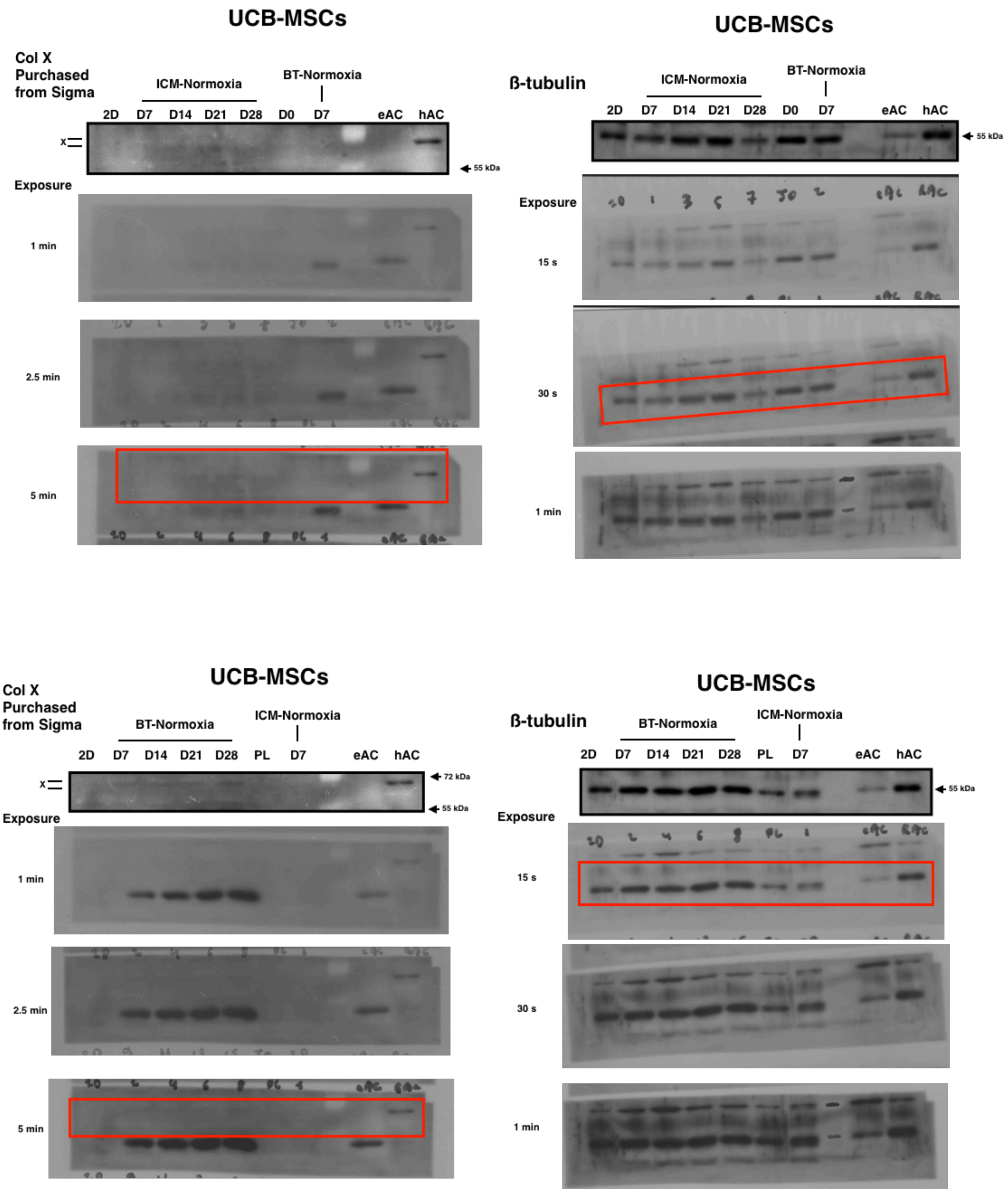

Supplemental figure 19: Complete gel and the polyvinylidene difluoride membranes analyzed in the western-blots.

For the western-blots presented in supplemental figure 2 (bottom WB for UCB-MSCs), the blots were obtained from 1 independent experiment and 1 western-blot of 2 gels. The cropped images are highlighted in the red lines.

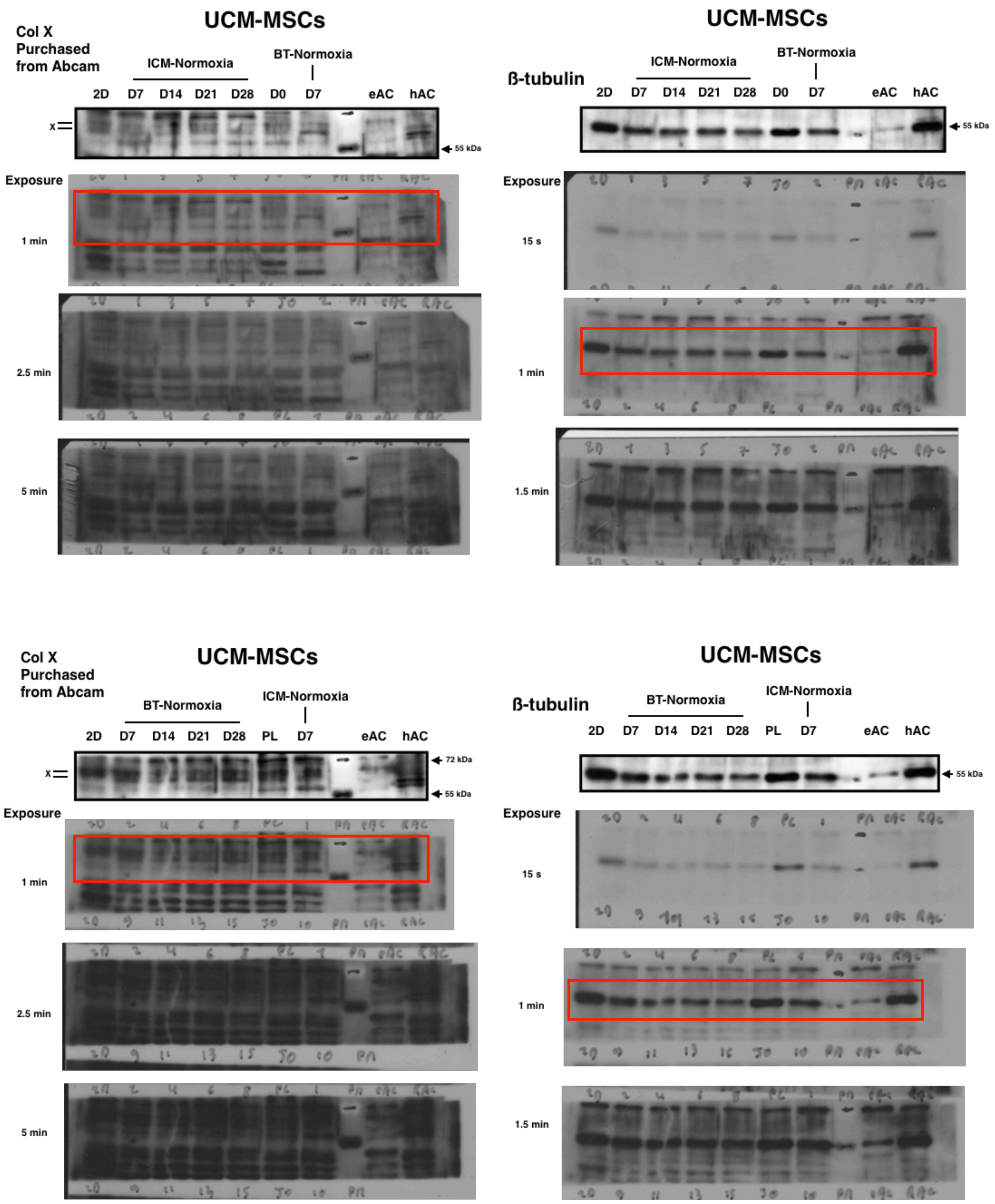

Supplemental figure 20: Complete gel and the polyvinylidene difluoride membranes analyzed in the western-blots.

For the western-blots presented in supplemental figure 2 (top WB for UCM-MSCs), the blots were obtained from 1 independent experiment and 1 western-blot of 2 gels. The cropped images are highlighted in the red lines.

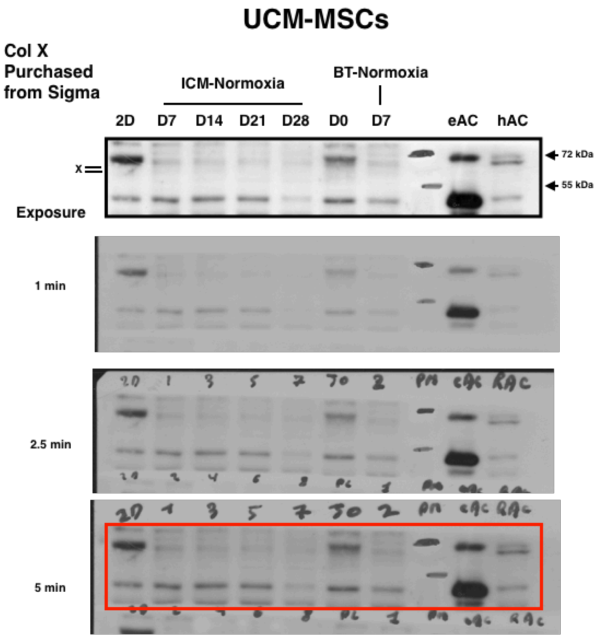

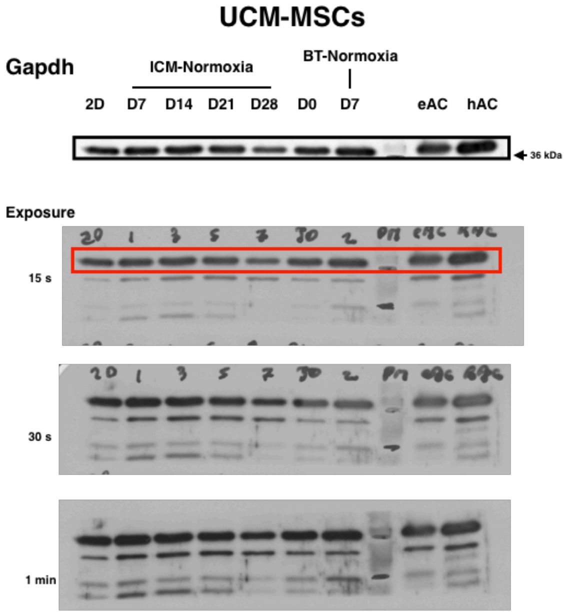

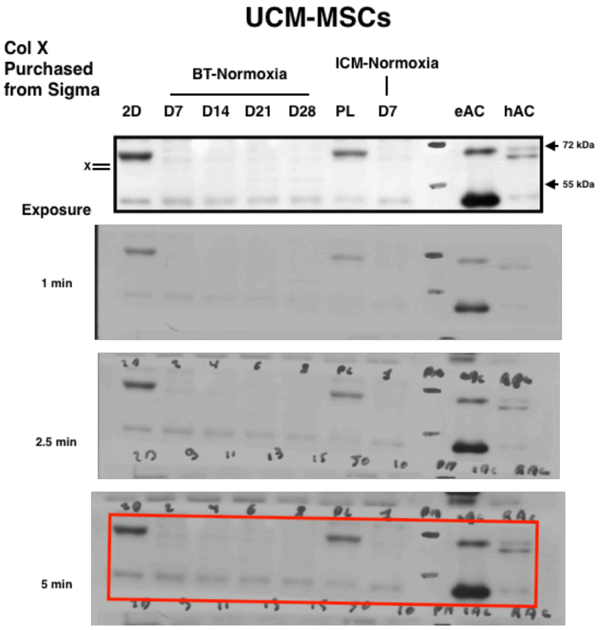

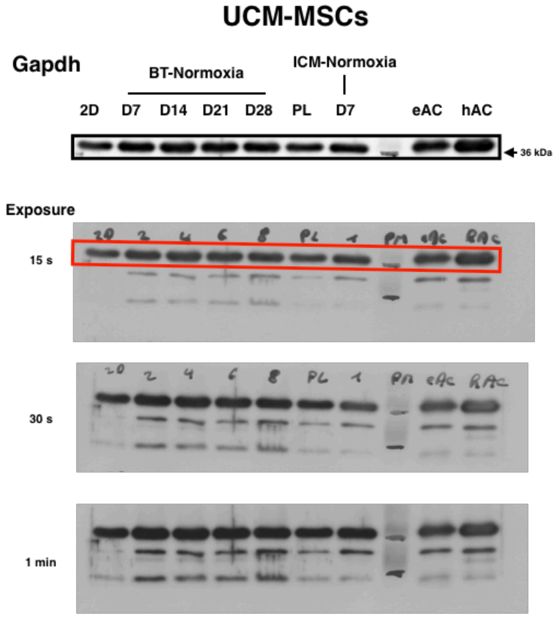

Supplemental figure 21: Complete gel and the polyvinylidene difluoride membranes analyzed in the western-blots.

For the western-blots presented in supplemental figure 2 (bottom WB for UCM-MSCs), the blots were obtained from 1 independent experiment and 1 western-blot of 2 gels. The cropped images are highlighted in the red lines.

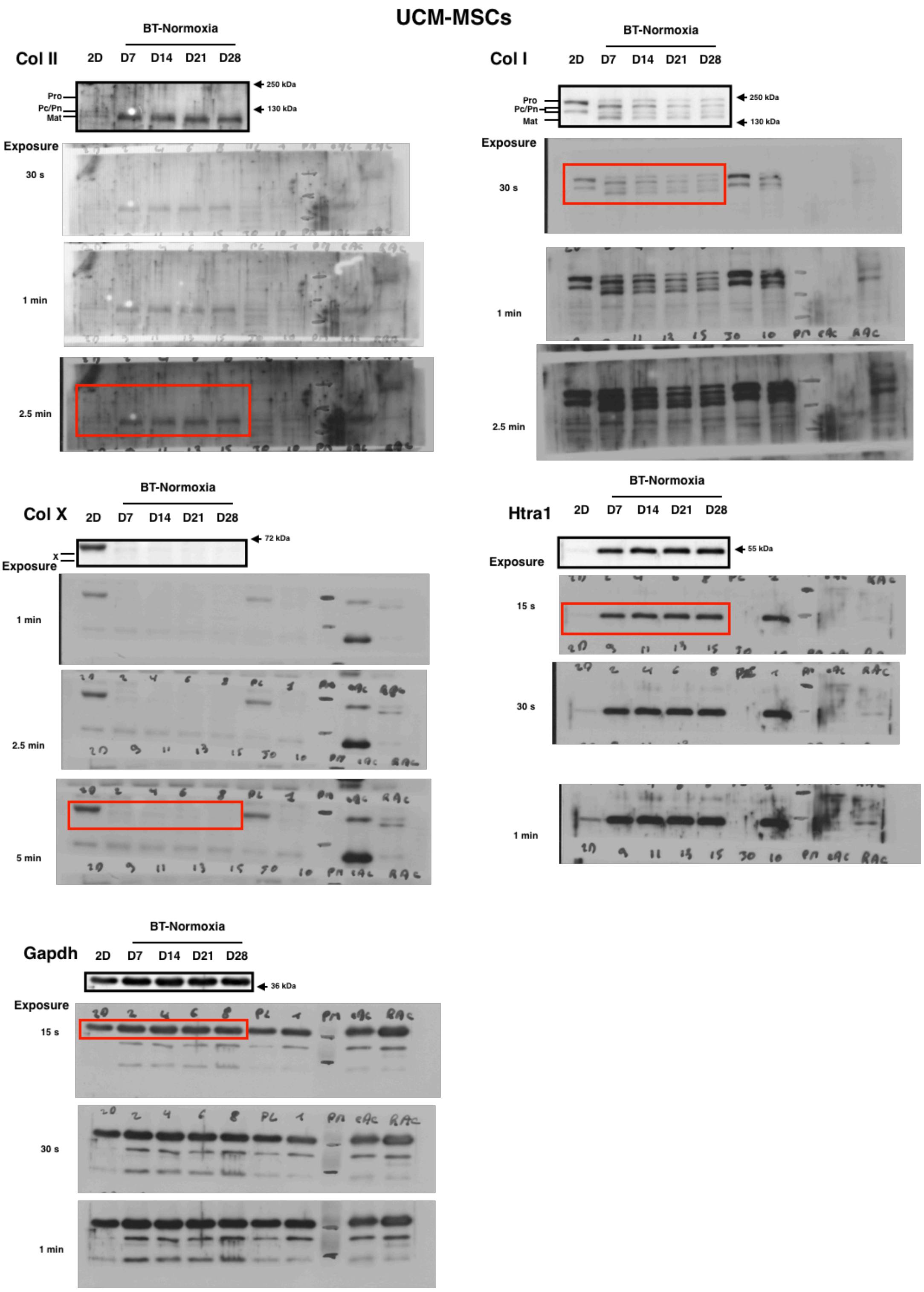

Supplemental figure 22: Complete gel and the polyvinylidene difluoride membranes analyzed in the western-blots.

For the western-blots presented in supplemental figure 5 (left), the blots were obtained from 1 independent experiment and 1 western-blot of 1 gel. The cropped images are highlighted in the red lines.

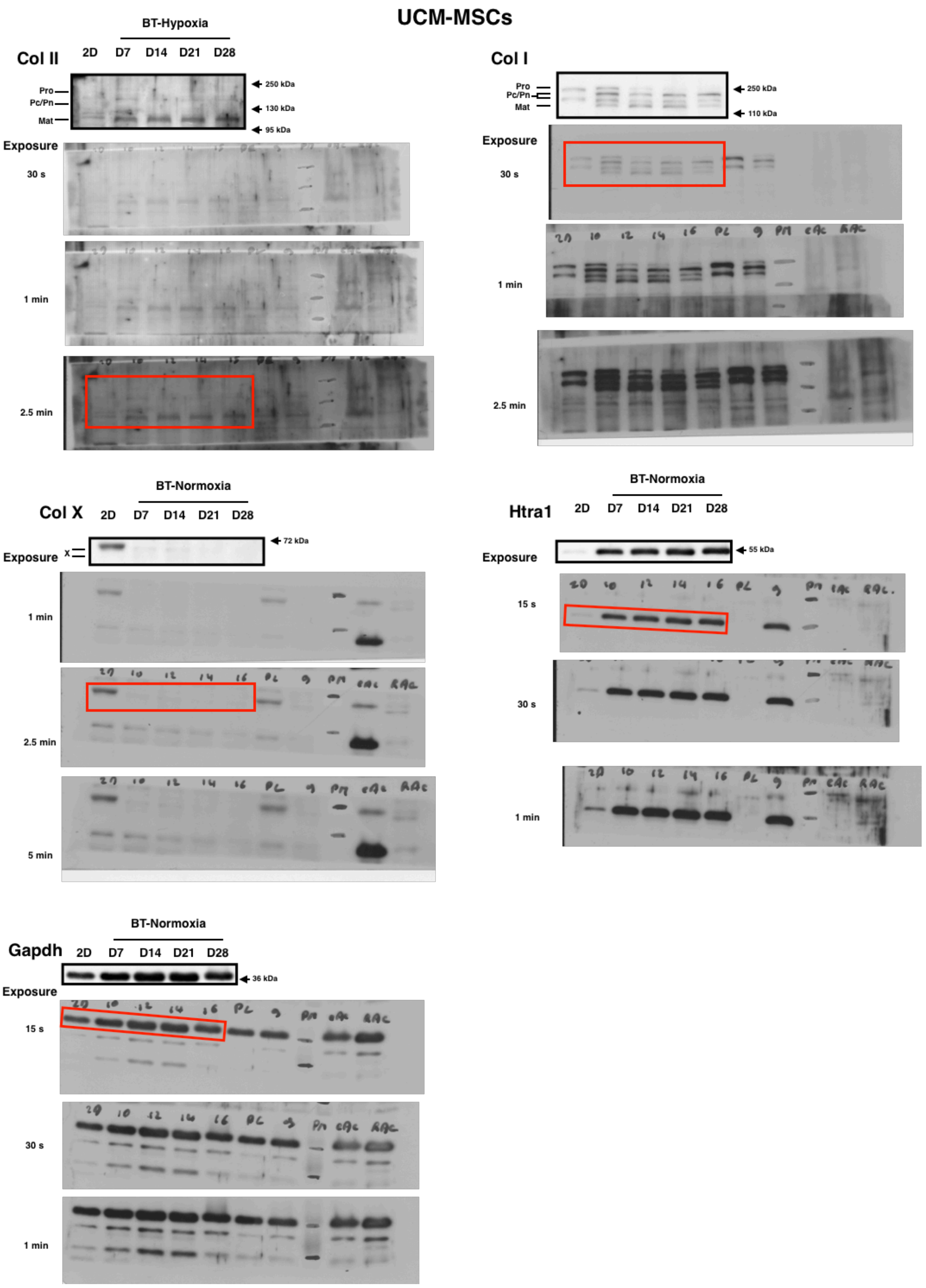

Supplemental figure 23: Complete gel and the polyvinylidene difluoride membranes analyzed in the western-blots.

For the western-blots presented in supplemental figure 5 (right), the blots were obtained from 1 independent experiment and 1 western-blot of 1 gel. The cropped images are highlighted in the red lines.
